# Multiple chromosomal rearrangements in a hybrid zone between *Littorina saxatilis* ecotypes

**DOI:** 10.1111/mec.14972

**Published:** 2019-02-25

**Authors:** Rui Faria, Pragya Chaube, Hernán E. Morales, Tomas Larsson, Alan R. Lemmon, Emily M. Lemmon, Marina Rafajlović, Marina Panova, Mark Ravinet, Kerstin Johannesson, Anja M. Westram, Roger K. Butlin

**Affiliations:** ^1^ Department of Animal and Plant Sciences University of Sheffield Sheffield UK; ^2^ Department of Marine Sciences, Centre for Marine Evolutionary Biology University of Gothenburg Gothenburg Sweden; ^3^ Department of Scientific Computing Florida State University Tallahassee Florida; ^4^ Department of Biological Science Florida State University Tallahassee Florida; ^5^ Department of Marine Sciences at Tjärnö, Centre for Marine Evolutionary Biology University of Gothenburg Strömstad Sweden; ^6^ Centre for Ecological and Evolutionary Synthesis University of Oslo Oslo Norway; ^7^ IST Austria Klosterneuburg Austria

**Keywords:** balancing selection, Gastropoda, inversion, linkage disequilibrium, local adaptation, recombination suppression

## Abstract

Both classical and recent studies suggest that chromosomal inversion polymorphisms are important in adaptation and speciation. However, biases in discovery and reporting of inversions make it difficult to assess their prevalence and biological importance. Here, we use an approach based on linkage disequilibrium among markers genotyped for samples collected across a transect between contrasting habitats to detect chromosomal rearrangements de novo. We report 17 polymorphic rearrangements in a single locality for the coastal marine snail, *Littorina saxatilis*. Patterns of diversity in the field and of recombination in controlled crosses provide strong evidence that at least the majority of these rearrangements are inversions. Most show clinal changes in frequency between habitats, suggestive of divergent selection, but only one appears to be fixed for different arrangements in the two habitats. Consistent with widespread evidence for balancing selection on inversion polymorphisms, we argue that a combination of heterosis and divergent selection can explain the observed patterns and should be considered in other systems spanning environmental gradients.

## 1 INTRODUCTION

The potential roles of chromosomal rearrangements in adaptation and speciation have been investigated almost since their discovery, approximately a century ago (Dobzhansky, 1970; Sturtevant, 1926, 1938). However, their contributions to these processes remained poorly understood until attention was given to their effects on recombination, especially the suppression of recombination in heterozygotes (Faria & Navarro, 2010; Rieseberg, 2001; Trickett & Butlin, 1994).

When speciation requires the build‐up of associations among traits involved in reproductive isolation in the face of gene flow, genetic architectures that suppress recombination between loci involved in these traits are likely to evolve (Smadja & Butlin, 2011). This is the case for chromosomal rearrangements, including inversions, translocations and fusions/fissions. Here, we focus on inversions where effective recombination is severely reduced or even completely suppressed in heterozygotes for two arrangements (i.e., heterokaryotypes), particularly near breakpoints (Coyne, Meyers, Crittenden, & Sniegowski, 1993; Navarro, Betrán, Barbadilla, & Ruiz, 1997; Sturtevant 1921; Sturtevant & Beadle, 1936, Schaeffer et al., 2003). It has been claimed that the recombination‐suppression effect of inversions can contribute to adaptation and speciation with gene flow in various ways: (a) extending the impact of barrier loci (i.e., loci contributing to reproductive isolation) to linked loci over wider genomic regions and facilitating the accumulation of additional barrier loci within inverted regions despite gene flow between populations (Navarro & Barton, 2003; Rieseberg, 2001), (b) preventing species merging after secondary contact and so paving the way for the accumulation of additional reproductive barriers (e.g., by reinforcement) (Noor, Grams, Bertucci, & Reiland, 2001) and (c) protecting favourable combinations of locally adapted alleles from being lost, including stochastic loss (Kirkpatrick & Barton, 2006; Rafajlovic, Emanuelsson, Johannesson, Butlin, & Mehlig, 2016) or maintaining combinations of alleles that contribute to different barriers, including assortative mating and incompatibilities (Dagilis & Kirkpatrick, 2016; Ortiz‐Barrientos, Engelstädter, & Rieseberg, 2016). A prediction underlying these different roles is that, in the presence of gene flow, inversions will tend to be enriched for barrier loci.

Empirical data from an increasing number of taxa support the role of inversions in adaptation and speciation (Hoffmann & Rieseberg, 2008; Hooper & Price, 2017; Wellenreuther & Bernatchez, 2018), although for historical reasons much of the evidence concerning the evolutionary genetics of inversions still comes from one genus; *Drosophila *(Dobzhansky & Sturtevant, 1938; Krimbas & Powell, 1992). However, the power to detect the genomic regions involved in adaptive traits and/or reproductive isolation is generally higher within rearrangements. This is because the effects of selection extend to linked sites across large regions of the genome, thus increasing the probability of detection by genome scans (Ravinet et al., 2017) and potentially biasing evidence in favour of inversions. On the other hand, studies showing that adaptation and speciation in some taxa are not influenced by inversions (e.g., Davey et al., 2017; Rafati et al., 2018) may receive less attention than those with positive results. In order to achieve an unbiased view of the occurrence and impacts of inversions, approaches are needed that allow for the detection of inversions without relying on pre‐existing information either from cytogenetic evidence, which remains limited to taxa where high‐resolution chromosome preparations can be obtained, or from genome scans for differentiation.

Hybrid zones offer a singular setting for investigating the genomic regions involved in reproductive isolation between natural populations (Barton & Hewitt, 1985; Harrison, 1993; Harrison & Larson, 2016). Classic hybrid zone theory predicts that alleles at loci under divergent selection or loci involved in incompatibilities introgress less compared with other markers (Barton & Hewitt, 1985; Rieseberg, Whitton, & Gardner, 1999). This results in clines in allele frequency with the slope at the cline centre, relative to dispersal distance, increasing with the intensity of selection (Barton & Gale, 1993; Barton & Hewitt, 1985; Slatkin, 1973). Inversions may be favoured by selection on one side of the hybrid zone because they may keep together combinations of locally adapted alleles at different loci, preventing or severely reducing recombination with migrant haplotypes from generating less fit individuals (Kirkpatrick & Barton, 2006). Previous studies of hybrid zones between taxa differing by inversions, translocations or fusions, have revealed greater differentiation at neutral markers in genomic regions within or near chromosomal rearrangements, suggesting a barrier to gene flow (Giménez et al., 2017; Lee et al., 2017; Rieseberg et al., 1999). Altogether, this suggests that hybrid zone studies can provide useful information about the presence of chromosomal rearrangements and their role in adaptation and speciation.

Although hybrid zones have been extensively studied, the opportunity that they provide to detect rearrangements de novo using genome‐wide markers has not been widely exploited (see Lee et al., 2017 and Westram et al., 2018 for exceptions). A wide variety of genotypes is produced by recombination in the central part of a hybrid zone, but linkage disequilibrium (LD) is continuously generated by dispersal (Barton & Hewitt, 1985). Inverted regions with suppressed recombination are expected to alter the balance between these forces, generating blocks of LD that stand out against the genomic background. In a sample taken from a transect across a hybrid zone, LD will also be generated by differentiation between parental populations but the loci involved are expected to be spread across the genome, rather than gathered in blocks. Therefore, patterns of LD among loci enable the de novo detection of inversions. Importantly, the same data can then be used to estimate inversion clines, allowing simultaneous assessment of their role in divergence. Candidate rearrangements can be validated by complementary approaches (e.g., linkage maps, genome synteny, BAC‐FISH). The sequence of events building up associations between adaptive alleles and inversions is not yet well known for most case studies (Jackson, Butlin, Navarro, & Faria, 2016) and hybrid zone studies may help here as well.

The rocky intertidal encompasses steep gradients of several factors (e.g., wave exposure, temperature, salinity, humidity, predation, competition and facilitation; Raffaelli & Hawkins, 1996), providing a fertile ground to improve our understanding of adaptation and the origins of reproductive isolation. The presence of locally adapted distinct ecotypes in the intertidal has been investigated in several gastropod species (*Nucella lapillus*, *Littorina saxatilis* and *L. fabalis*; Johannesson et al., 2010; Reimchen, 1981; Rolán‐Alvarez, Austin, & Boulding, 2015; Rolán, Guerra‐Varela, Colson, Hudges, & Rolán‐Alvarez, 2004; Rolán & Templado, 1987; Tatarenkov & Johannesson, 1998), and also suggested in *L. arcana*, *L. compressa*, *L. striata* and *Melarhaphe neritoides* (Garcia, Pérez Diz, Sá‐Pinto, & Rolán‐Alvarez, 2013; Reid, 1996). Among these species, the rough periwinkle (*Littorina saxatilis*) comprises one of the best‐characterized examples of parallel evolution of two divergent ecotypes (“Crab” and “Wave”) across different geographic regions (e.g., Spain, Sweden and the UK) facing similar selective pressures (mainly crab predation and wave exposure) (Butlin et al., 2014). In many locations across the species range, the two ecotypes meet at steep environmental transitions (on scales ~10 m). Parallel divergence between ecotypes involves multiple phenotypic traits (e.g., shell thickness, shell size, shell shape, shell colour and boldness) (Johannesson et al., 2010), and multiple loci (Westram, Panova, Galindo, & Butlin, 2016). This provides a setting in which suppressed recombination within inverted regions could play an important role in protecting favourable combinations of alleles at different loci, fostering adaptation to a multidimensional environment.

Sequencing approaches targeting loci putatively influenced by divergent selection (i.e., outliers; Galindo, Grahame, & Butlin, 2010; Ravinet et al., 2016; Westram et al., 2014) suggest a partly shared genetic basis, mainly at local geographic scales (Westram et al., 2016). Despite the identification of multiple genomic regions likely to contain barrier loci between ecotypes, until recently the genetic architecture of ecotype divergence and speciation in *L. saxatilis* remained unknown. Low LD in a hybrid zone in the UK suggested that outlier loci were dispersed in the genome (Grahame, Wilding, & Butlin, 2006). However, resources now available for this species, including a reference genome and a genetic map for the Crab ecotype (Westram et al., 2018), have altered this picture. A study of a hybrid zone between *L. saxatilis* ecotypes in Sweden, using targeted resequencing of approximately 40,000 regions of the genome revealed a large number of SNPs (1,891) with clinal patterns that are not compatible with neutral expectations (based on system‐specific simulations), suggesting the influence of divergent selection. Remarkably, ~75% of these SNPs (non‐neutral or linked to non‐neutral loci) were shown to be clustered in large genomic regions of high LD (12.5–29.5 cM) in three out of 17 linkage groups (putative chromosomes), suggesting large regions of low recombination compatible with the presence of chromosomal rearrangements (Westram et al., 2018). Finally, rare fixed differentiation between ecotypes, combined with steep clines, at many of these loci led Westram et al. (2018) to suggest a component of balancing selection, rather than purely divergent selection. Interestingly, balancing selection has frequently been documented for inversion polymorphisms (e.g., Butlin & Day, 1985; Dobzhansky, 1950; reviewed by Wellenreuther & Bernatchez, 2018).

Using cytogenetic techniques, the karyotype of *L. saxatilis* has been established, with a haploid number of 17 chromosomes that appears to be conserved among ecotypes and closely related species (Birstein & Mikhailova, 1990; Janson, 1983; Rolán‐Alvarez, Buño, & Gosalvez, 1996). However, the poor resolution of these techniques did not allow for identification of chromosomal rearrangements. Here, we combine genomic resources and genetic data from laboratory crosses for *L. saxatilis* with LD information from a hybrid zone. We test the proposal that the genomic blocks of outlier SNPs detected by Westram et al. (2018) correspond to inversions and we survey the rest of the genome for additional polymorphic inversions. For all putative inversions detected, we examine arrangement frequency clines in order to reveal evolutionary forces shaping these polymorphisms.

## 2 MATERIALS AND METHODS

We reused a data set published by Westram et al. (2018), consisting of SNPs derived from targeted resequencing of individuals from a *L. saxatilis* hybrid zone transect, a reference genome assembly and a linkage map generated for a Crab‐ecotype family. We add similar resequencing data from four families of the Wave ecotype.

### 2.1 Data from Westram *et al.* (2018)

**Figure 1:**
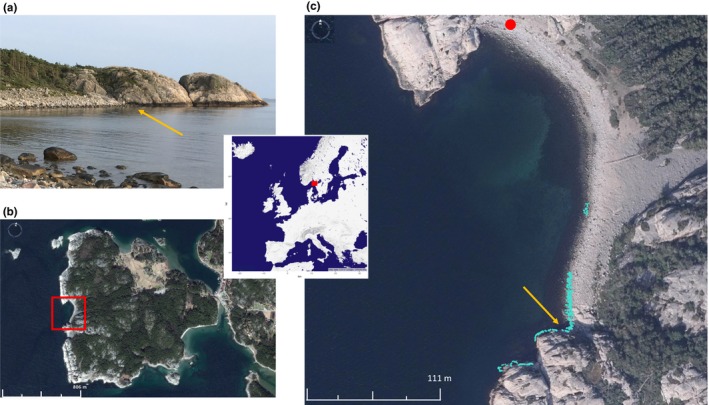
(a) Photograph of the sampled shore area showing the typical habitat of the Crab (boulders) and Wave ecotypes (bedrock), taken from the red circle in (c). (b) Map of the sampling region with the red square enclosing the area shown in (c). (c) Sampling transect across the two habitats with the position of the sampled snails (cyan) (data from Westram et al., 2018, image from www.Hitta.se). Map of Europe is shown at the centre with a red square marking the sampling region on the west coast of Sweden. Map and satellite image in (b) were obtained from Google Earth (Image© 2018 DigitalGlobe). Orange arrows point to the major habitat transition

Snails were collected in Sweden (Ängklåvebukten; N 58° 52' 15.14", E 11° 7' 11.88") across a 152‐m transect along the shore and their positions in three dimensions was recorded (Figure 1). The transect spanned an environmental gradient from a boulder field to a cliff area, the typical habitats of the Crab and Wave ecotypes, respectively (Figure 1a,b). After DNA extraction from 373 individuals using a CTAB protocol (Panova et al., 2016), a targeted‐capture sequencing approach was implemented using 120‐bp probes designed for 40,000 regions in the *L. saxatilis *genome. After library preparation, sequencing was performed using an Illumina HiSeq 2000 platform. A custom bioinformatics pipeline was implemented, including steps for stringent quality control (for details see Westram et al., 2018). A final set of 44,251 variants was later used in the linkage disequilibrium and principal component analyses. Individuals with more than 50% of missing data were removed.

In addition, we made use of two other key resources from Westram et al. (2018): (a) a reference genome generated for a Crab individual (388,619 scaffolds/contigs, N50 scaffolds of 40,374 bp, NG50 of 55,450 bp), and (b) a linkage map for one full‐sib Crab family (186 offspring) generated with Lep‐Map2 (Rastas, Calboli, Guo, Shikano, & Merilä, 2016), based on the same capture approach and bioinformatics procedures as described above, resulting in 18,942 markers (total map length of 1,011.9 cM with a resolution of ~0.5 cM) distributed across 17 linkage groups (LGs). The number of LGs corresponds to the haploid number of chromosomes described for *L. saxatilis* (Birstein & Mikhailova, 1990; Janson, 1983; Rolán‐Alvarez et al., 1996).

### 2.2 Genotyping of Wave families

In order to infer recombination in the Wave ecotype, juvenile virgin females were collected from a wave‐exposed habitat at Ängklåvebukten (north end) and kept in separate aquaria with running seawater. At the time of female maturity (9 months later), adult males were collected from the same area and paired with the females. Crosses resulted in four full‐sib families (8, 21, 12 and 11 offspring). Although a different female was used in each cross, the first three families shared the same father. Genotyping of one female parent failed so that only three families were available for analysis of female‐informative markers.

Targeted resequencing was performed using the same targeted‐capture sequencing approach but using about half of the probe set used in Westram et al. (2018). We preferentially retained informative probes and avoided probes close together within contigs. In total, 25,000 (120 bp) enrichment probes were used. Following Lemmon, Emme, and Lemmon (2012), indexed libraries were prepared for 58 individuals (52 offspring, 4 mothers and 2 fathers) from genomic DNA on a Beckman Coulter FXp liquid‐handling robot, and enriched using an Agilent SureSelect enrichment kit at Florida State University's Center for Anchored Phylogenomics (www.anchoredphylogeny.com). Following qPCR and Bioanalyzer‐based quality control, libraries were sequenced on a partial Illumina 2,500 lane with paired‐end 150‐bp reads and 8‐bp indexing read.

Raw reads were cleaned with trimmomatic v. 0.36 (Bolger, Lohse, & Usadel, 2014) with default parameters for paired‐end reads and quality confirmed with fastqc v0.11.5 (Andrews, 2010), resulting in the removal of three samples due to low quality. Cleaned reads were mapped to the *L. saxatilis* reference genome using bwa v0.7.15 (Li & Durbin, 2009), retaining all probe regions that were covered by at least five reads in at least 50% of samples. Since the probes cover only a subset of the reference genome, contigs with lower coverage or not included in the probe design were merged into a single “superscaffold” to reduce computational time for SNP calling. Reads were again mapped using bwa to this new reference genome.

PCR duplicates were identified and removed, and InDel realignment performed with piccard v. 1.138 (http://broadinstitute.github.io/picard/ ), before SNP calling, which was performed using gatk unifiedgenotyper v3.7‐0 (DePristo et al., 2011) with default parameters and a minimum base quality filter of 20. The SNP calling was restricted to the better‐covered probe region using a bed file, ignoring the entire “superscaffold” region. We removed positions and individuals with <25% call rate and retained only biallelic SNPs then used technical replicates to train a variant quality score recalibration model in order to improve parameter values for SNP calling. Lastly, we used hard‐filters (mapping quality >40, Phred‐scaled *p*‐value for strand bias <10, symmetrical odds ratio test for strand bias <3 and test for read position bias between 0 and 8.0) and only retained SNPs with coverage depth ≥8. The SNP filtering workflow was performed with vcftools (Danecek et al., 2011) and vcfilter from vcflib (https://github.com/vcflib/vcflib). This set of SNPs was filtered using the criteria in Westram et al. (2018), with minor allele frequency >0.05 and excluding sites with genotypes for fewer than 20 out of 55 individuals). A genotype file for the final set of SNPs (34,787) was generated using vcftools and was used as input for the recombination analysis.

### 2.3 Linkage disequilibrium

We analysed patterns of disequilibrium among SNPs in order to detect clusters of loci with unusually high LD that might be generated by chromosomal rearrangements. A matrix of pairwise LD (*r* ^2^) between all SNPs within each linkage group was generated for all individuals in the transect sample with the r package “genetics” (Warnes, Gorjanc, Leisch, & Man, 2013). This matrix was then used to detect clusters of SNPs in high LD (i.e., outlier clusters relative to other LD clusters within each linkage group) using the r package “ldna”—linkage disequilibrium network analysis (Kemppainen et al., 2015). Two key parameters can be set by the user to make the analyses more lenient or conservative in the identification of outlier clusters (OC). The minimum number of edges |E|min, corresponds to the minimum number of connections among the vertices (SNPs) of a cluster (an “edge” is present between a pair of SNPs if their LD value exceeds a threshold), and indirectly controls the minimum number of SNPs within a cluster. Parameter φ controls the minimum LD threshold above which the median pairwise LD within a cluster is higher than the intercluster LD for the group of SNPs to be considered an OC. After several test runs, we set |E|min = 30, representing a compromise between detecting clusters large enough to represent chromosomal rearrangements and avoiding noise created by small networks that result from physical linkage within contigs. As in most cases the number of edges did not correspond to the number of SNPs, only clusters with a minimum of 32 SNPs were retained. In order to explore a wide range of the parameter space of *φ*, we first registered all identified clusters with at least 32 SNPs setting *φ* = 0 and then increased the value of *φ* by 1 in each iteration until no more LD clusters were obtained within a linkage group. Given that chromosomal rearrangements are expected to generate strong LD, clusters with a low median intracluster LD (*r* ^2^ < 0.3) were also discarded. Whenever clusters obtained for the different values of *φ* shared SNPs, the one with the smaller number of SNPs (and higher median LD) was retained. The only two exceptions occurred when SNPs from two overlapping clusters became fused into a single larger cluster at higher *φ*, suggesting a common source of LD. In these cases, only the merged larger cluster was retained for downstream analyses. Although LDna allows detection of two different types of clusters, single‐outlier clusters (SOCs) and compound‐outlier clusters (COCs), the latter were disabled as they can be generated by different evolutionary forces acting simultaneously, making the interpretation of results difficult (Kemppainen et al., 2015). The final lists of SOCs for each linkage group (LGCs) and their sizes (the map distance between the coordinates of the most extreme positions of the SNPs included in each SOC, according to the Crab linkage map) were then investigated in the downstream analyses.

### 2.4 Principal component analysis (PCA)

ldna can detect clusters of loci that are in LD for various different reasons, primarily the effects of inversions (or other chromosomal rearrangements) on recombination, spatial population structure, or structure generated by local adaptation. Kemppainen et al. (2015) suggest that LD clusters due to inversion polymorphism can be identified because the SNPs involved are genomically clustered and they identify groups of genetically distinct individuals that correspond to different karyotypes. Within an inversion segregating in a population, we expect that suppressed recombination between arrangements will result in the presence of three distinct groups of individuals (homokaryotypes for the reference arrangement, heterokaryotypes, and homokaryotypes for the alternative, inverted arrangement). Allele frequencies at many SNP loci are expected to differ between arrangements because of their partly independent evolution.

Kemppainen et al. (2015) illustrated how the different genotypic groups could be separated in principal component analysis of SNPs within an LD cluster, generating a characteristic pattern in which the group of heterokaryotype individuals falls between two groups of homokaryotypes on PC1, because of their intermediate allelic content. Note that if three alternative chromosomal arrangements are present in the same genomic region, there will be three groups of homokaryotype individuals (AA, BB and CC) and three heterokaryotype groups (AB, BC and AC) and they are expected to form a triangle on a PC1 vs. PC2 plot with the homokaryotypes at the vertices. Therefore, we performed PCA using the r package pcadapt (Luu, Bazin, & Blum, 2017) for each SOC within each linkage group, using all the SNPs within the coordinates (not just those in high LD that led to identification of the SOC). For comparison, we also ran a PCA for the SNPs within the same linkage group outside the SOC coordinates, that is, within putatively collinear regions. The composition of groups of genotypes was then identified using the r function “kmeans,” which clusters data based on similarity using the algorithm developed by Hartigan and Wong (1979). The number of groups was set to three, or six when two SOCs presented overlapping coordinates, suggesting two putative rearrangements and so the possibility of three haplotypes. Since different groups can be obtained in different runs, each data set was analysed 10 times and we kept the run with the highest proportion of the sum of squares between clusters over the total. A single exception was observed for a SOC in linkage group LG14, where no resulting group reflected the observed structure in the data; in this case, groups were defined manually based on the position of individuals in the PCA plot (see below). Grouping was based on the first principal component, except in the case of overlapping SOCs, where the grouping algorithm was applied to the first and second components together. Only the SOCs showing absent or rare intermediate individuals between the three (or six) groups obtained in the PCA and with the first principal component explaining at least 10% of the variance, were kept as candidate inversions in downstream analysis, in order to restrict our analyses to low‐recombination regions with relatively high differentiation between genotypes.

### 2.5 Genetic diversity

If the groups detected in the PCA represent homo‐ and heterokaryotypic individuals for polymorphic inversions, then we expect the central group (heterokaryotypes) to have high heterozygosity relative to the more extreme groups (homokaryotypes) on PC1 (and PC2 where there are 6 groups) and relative to collinear regions of the same linkage group. This pattern is expected to be particularly marked for SNPs with strong allele frequency differences between arrangements. We tested this prediction for each candidate inversion, by calculating observed heterozygosity (*H*_obs_). Kemppainen et al. (2015) used this prediction to distinguish between LD clusters generated by inversions and those generated by population structure. In our case, this distinction is less clear‐cut since we expect an increase in heterozygosity in the centre of the transect and individuals from this region may also fall centrally on PC1. Observed heterozygosity for each variable position in the whole data set was estimated for each group identified by the PCA (the homokaryotypes for each candidate inversion arrangement and heterokaryotypes) using the “dfgenin” function of the r package “adegenet” (Jombart & Ahmed, 2011). The difference in *H*_obs_ between inverted and collinear regions within each LG was tested by means of Wilcoxon rank‐sum tests.

Whereas *H*_obs_ was used to test specifically the expectation that the individuals of the PCA groups corresponding to putative heterokaryotypes are heterozygous for many SNPs, we used nucleotide diversity (*π*) to assess the genetic variation present in each arrangement. For a young inversion, we expect one arrangement to have low *π* relative to the other arrangement and relative to collinear regions (outside LGCs). These differences should decrease with time due to mutation and gene flux (due to double crossovers and gene conversion; Stevison, Hoehn, & Noor, 2011) while divergence between arrangements (*d_XY_*) should increase. We calculated *π* and *d_XY_* to give a first view of the ages of inversions but note that other factors influence these statistics (see below). Nucleotide diversity was estimated for the two homokaryotypes of each LGC using vcftools in order to compare arrangements. *π* per site was estimated for each SNP and then averaged across all sites within the length of each probe region, including invariant sites (~120 bp). Pairwise divergence between the putative homokaryotypes (*d_XY_*) was estimated in the same way as *π* for each probe region, using the fact that *π_t_* (for a group containing both homokaryotypes) is based on a mixture of comparisons between and within karyotypes. Specifically,

$$N\left(N-1\right)\pi_{t}=2n_{x}n_{y}d_{\mathrm{XY}}+n_{x}\left(n_{x}-1\right)\pi_{x}+n_{y}\left(n_{y}-1\right)\pi_{y}$$

where *n_x_*and *n_y_*are the numbers of the two homokaryotypes, *N = n_x_ + n_y_*, and *π_x_* and *π_y_* are nucleotide diversities for the two homokaryotypes separately. Differences in *π* and *d_XY_* between putatively inverted and collinear regions (as control) within each LGC were examined using Wilcoxon rank‐sum tests. Finally, differences in *π* between the homokaryotypes for each LGC were also tested for inverted and noninverted regions (as control) using Wilcoxon rank‐sum tests. All tests were adjusted for multiple comparisons using the sequential Bonferroni correction.

### 2.6 Recombination patterns

The presence of inversion polymorphism can be confirmed by their effects on patterns of recombination (in our case, the realised crossover patterns detected in offspring). Two effects may be observed in crosses: suppression of recombination in inverted regions where the informative parent is a heterokaryotype, and reversal of part of the genetic map when comparing parents that are homokaryotypes for opposite arrangements. To test these predictions, we re‐examined the Crab‐ecotype linkage map of Westram et al. (2018) and we also compared recombination events in four Wave ecotype families among informative parents and with the predictions from the Crab map. There were insufficient individuals in the Wave families for construction of an independent Wave ecotype map.

For the Wave families, we performed a PCA followed by kmeans clustering including all samples from the hybrid zone and laboratory crosses to infer the karyotype of each parent. By comparing parent and offspring genotypes, SNPs with genotyping errors as well as with extreme segregation distortion were removed. Moreover, apparent recombination events involving only single SNPs, or multiple consecutive SNPs within the same contig, were removed because genotyping errors cannot be excluded in these cases. Thus, the number of recombination events in our data is conservative, regardless of the region (inverted or not) where they were detected.

**Figure 2:**
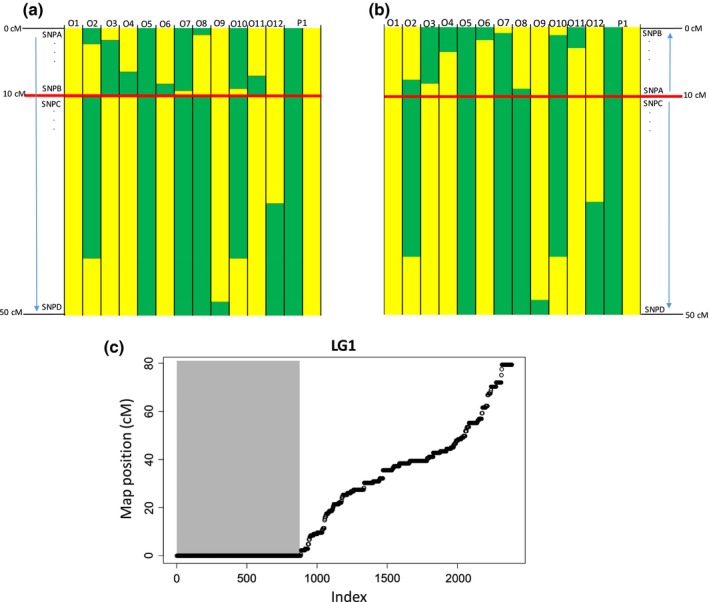
(a, b) Hypothetical recombination patterns supporting the presence of an inversion. Each column represents a Wave offspring multilocus haplotype (O1 to O12) and the two haplotypes for the informative parent in two different colours (P1). The other parent haplotypes are not represented as they would have the same colour in all individuals (i.e., not informative). Any switch from yellow to green within an individual represents a recombination event. In (a), markers are ordered according to the Crab map. In (b), markers in the inversion (above the red line) were reversed in order, representing the alternative arrangement. Individuals 2, 3, 4, 6, 7, 8, 10 and 11 are inferred to have double crossovers in (a) (reference gene order) but only single crossovers in (b) (alternative gene order). Thus, the inverted gene order is more parsimonious. (c) Mapping positions of markers on LG1 for one of the Crab parents (from Westram et al., 2018). The large number of markers with zero recombination in the region of LGC1.1 (grey) supports recombination suppression in this heterokaryotype parent. Index indicates the rank of the marker, by map position

We considered only male‐informative or female‐informative markers (not those heterozygous in both parents). For each parent separately, we then manually determined the parental haplotypes using informative SNPs and identified recombination events as positions where the haplotype switched in an offspring individual (Figure 2). We tested the expectation of suppressed recombination in heterokaryotype parents by counting recombination events and comparing to recombination events in collinear regions and in homokaryotype parents. We did the haplotype switching analysis using the order of SNPs inferred from the Crab family (reference arrangement) and then, where the parent was inferred to be an alternative homokaryotype, we reversed the gene order in the proposed inverted region in order to compare the pattern of recombination. We tested for patterns of recombination that were more likely under the alternative (Wave) arrangement. Specifically, offspring haplotypes that can only be generated by two crossover events given one arrangement can be caused by a single crossover under the alternative arrangement (Figure 2a,b). Finally, suppressed recombination within inversions where the Crab parents included a heterokaryotype was inferred from long map distances in one parent without recombination in the other parent (Figure 2c) or from large numbers of SNPs at a single map position in both parent‐specific maps.

### 2.7 Cline‐fitting

In order to investigate the possibility of selection acting on the inversions, we considered their distribution along the transect from Crab to Wave habitat. After classifying individuals according to the number of copies of the alternative arrangement (0, 1 or 2) for each inversion, based on the PCA clusters, changes in arrangement frequency across the transect were modelled as constant or clinal. Two cline models (a four‐parameter sigmoid cline, following equations in Derryberry, Derryberry, Maley, and Brumfield (2014), and a five‐parameter asymmetrical cline) were fitted using maximum likelihood (bbmle package in r, function mle2, Bolker (2012)). The symmetrical cline had parameters for centre, width, frequency in the Crab ecotype and frequency in the Wave ecotype. The asymmetrical cline had two width parameters, one for the Crab side of the centre and one for the Wave side. Widths were fitted after log transformation and allele frequencies after logit transformation, to avoid boundary effects. The best model was selected using Akaike's information criterion (∆AIC > 10). An additional criterion for conformity with clinal variation was that the proportion of genetic variation explained by the cline was >10% (measured as the deviance explained by the cline fit using a GLM with binomial error distribution). We tested whether one arrangement was fixed in one or the other ecotype by comparing the unconstrained cline fit (both end frequencies in the range 0,1) to a fit with the relevant frequency constrained to 0 or 1. A profile analysis was performed for the clinal inversions to test whether they shared the same centre or width. In this analysis, the sum of the log‐likelihoods for the best unconstrained fit for each inversion was compared to the best sum of log‐likelihoods for fits constraining the centre (or width) to one of a range of fixed values (from 85 to 100 m in 1 m steps for the centre and from 1 to 54.6 m, 0–4 in 0.2 steps on a log scale, for width). We tested the difference using 2∆LL = *χ* ^2^ with degrees of freedom equal to the number of inversions – 1 (i.e., the difference in number of parameters estimated).

## 3 RESULTS

### 3.1 Detection of candidate chromosomal rearrangements

**Figure 3:**
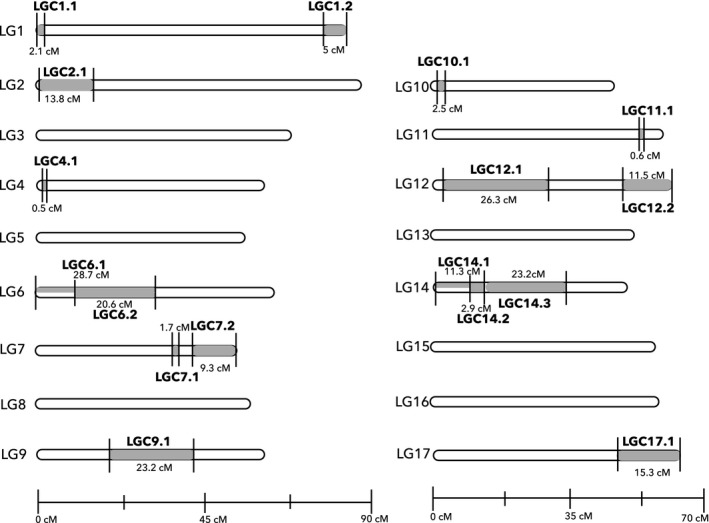
Distribution of the LD clusters (grey) identified with the network‐based analysis of LDna and filtered based on the PCA. Codes and approximate sizes of the candidate rearrangements are shown based on the information from Table 1. Linkage groups (white) were ordered based on the number of SNPs contained in each (high to low from LG1 to LG17, respectively). Part grey, part white indicates the overlapping inversions on LG6 and LG14

The implementation of the LD analyses followed by our filtering criteria resulted in the detection of 17 LD clusters of loci (SOCs) identified as the candidate chromosomal rearrangements that were then characterized in downstream analyses (Figure 3, Table 1).

**Table 1:** LD clusters comprising our 17 candidate rearrangements obtained with LDna and after filtering based on the PCA. Linkage groups, boundaries (with start and end positions according to the Crab linkage map produced by Westram et al. (2018)), numbers of SNPs in LD and their contigs, median LD between these SNPs as well as the variance explained by PC1 are shown

Linkage group (LG)	LD cluster	Cluster size (cM)	Start (cM)	End (cM)	Number of SNPs	Number of contigs	Median LD (r2)	PC1 variance (%)
LG1	LGC1.1	2.1	0	2.1	146	79	0.985	40
LG1	LGC1.2	5.42	75.53	80.95	34	22	0.970	28
LG2	LGC2.1	13.87	0.34	14.21	52	23	0.938	44
LG4	LGC4.1	0.48	1.03	1.51	145	67	0.947	33
LG6	LGC6.1^a^	29.30	0	29.30	135	54	0.397	47
LG6	LGC6.2^b^	20.57	8.73	29.30	100	35	0.613	42
LG7	LGC7.1	1.73	36.01	37.74	38	22	0.827	29
LG7	LGC7.2	9.29	42.08	51.37	32	15	0.79	22
LG9	LGC9.1	23.18	18.64	41.82	50	33	0.964	28
LG10	LGC10.1	2.54	0.58	3.12	76	41	0.938	25
LG11	LGC11.1	0.59	52.32	52.91	200	86	0.949	28
LG12	LGC12.1	26.31	3.32	29.63	37	21	0.442	14
LG12	LGC12.2	11.52	48.71	60.24	40	22	0.625	19
LG14	LGC14.1^a^	11.32	0.39	11.71	263	99	0.406	35
LG14	LGC14.2^b^	2.90	8.81	11.71	91	52	0.939	38
LG14	LGC14.3^b^	23.23	11.71	34.94	43	18	0.377	15
LG17	LGC17.1	15.33	46.99	62.32	81	35	0.91	50

a Variance explained by the PCA is relative to the first part of the LGC (nonoverlapping with other LGCs within the same LG).

b LD cluster identified by PC1 and PC2, with the latter explaining 17%, 16% and 6% of the variance for LGC6.2, LGC14.2 and LGC14.3, respectively.

Six LGs contained no LD cluster, 11 LGs contained at least one LD cluster and five of these contained at least two clusters. Each of these clusters was composed of SNPs spanning a single genomic region, their sizes varied between ~0.5 and 29.3 cM (coordinates based on the Crab linkage map) and they contained between 32 and 263 SNPs in relatively high LD (from median *r* ^2 ^= 0.377 to *r* ^2 ^= 0.985) distributed over 15–99 different contigs (Figure 3, Table 1). LD clusters containing distinct sets of SNPs but with overlapping map positions were detected on LG6 and LG14. We interpret this as the result of overlapping rearrangements and treat each component genomic region separately (Figure 3). Six out of the 17 LD clusters were located at the ends of LGs (although this may not mean that they were close to the physical ends of chromosomes because linkage mapping typically has difficulty in including markers at chromosome ends). Cluster 3 of LG14 (LGC14.3) and LGC12.1 showed the least support from PC1 (only 15% and 14% of the variance explained, respectively), whereas LGC17 showed the highest (50%).

**Figure 4:**
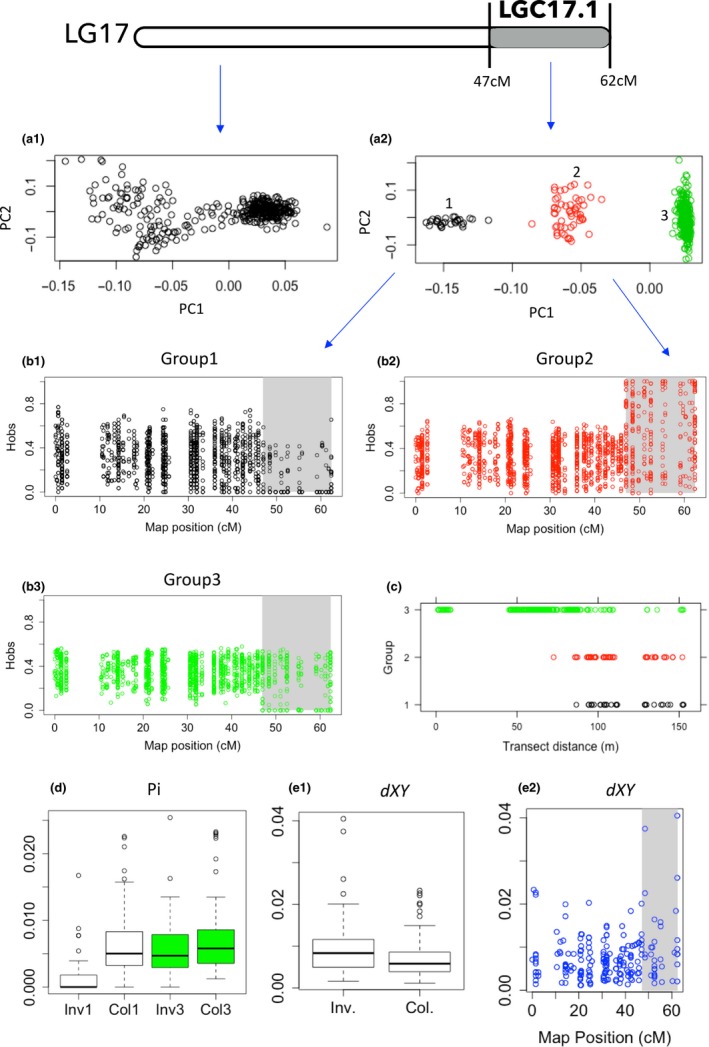
Characterization of LGC17.1 (grey). (a) PCA based on markers located in the collinear (a1) and putatively inverted region (a2). Three main groups were observed in the inverted region consistent with two homokaryotypes (black and green) and heterokaryotypes (red). (b) Observed heterozygosity across the genetic map for each of these groups (b1–b3). (c) Distribution of the three groups across the transect (distance from the Crab end is shown). (d) Boxplot of nucleotide diversity (pi) for the inverted and collinear regions of the groups 1 and 3. Divergence (*d_XY_*) between groups 1 and 3 within (Inv) and outside (Col) the inverted region (e1), as well as across the genetic map (e2)

**Figure 5:**
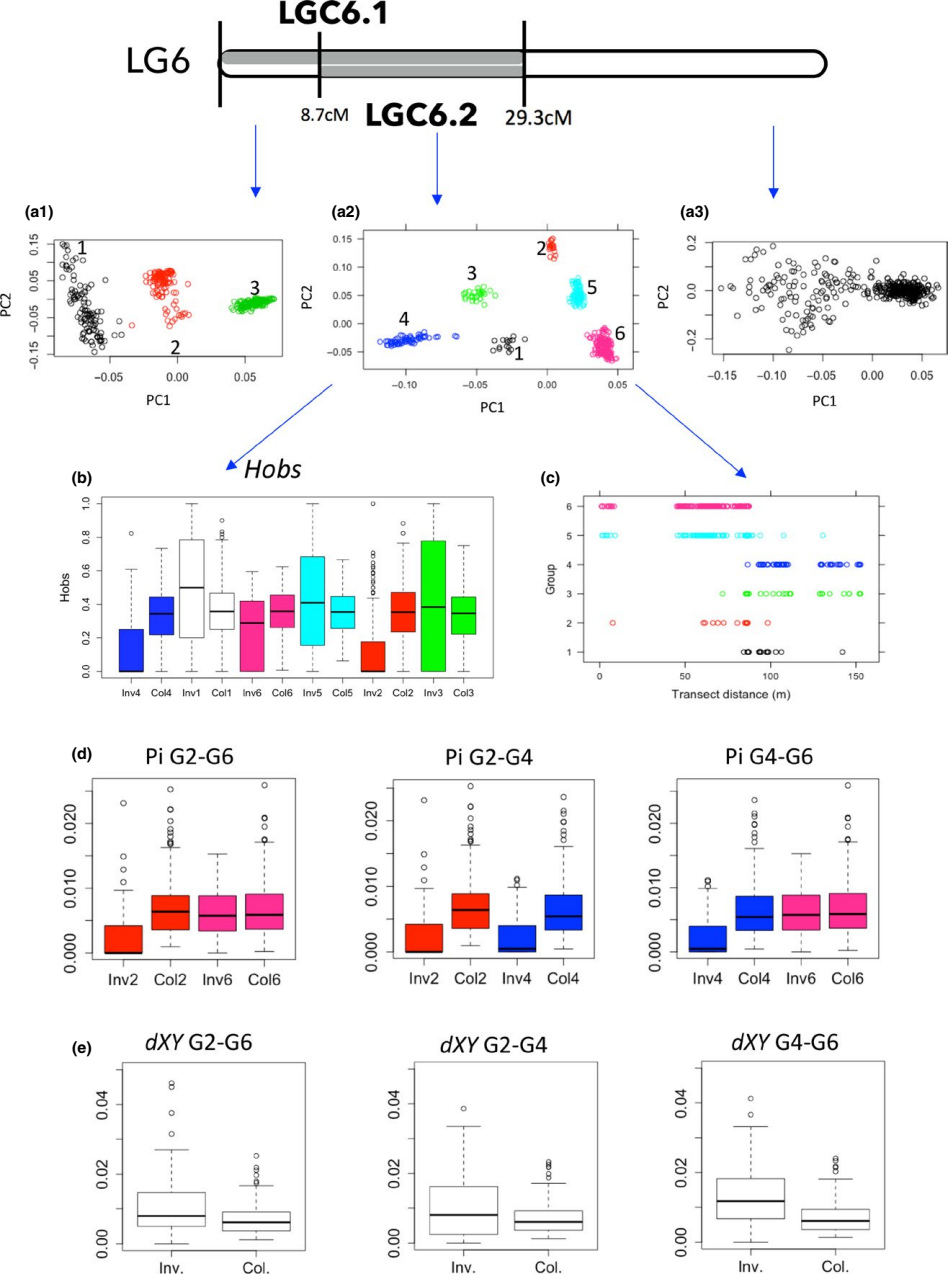
Characterization of LGC6.2 (smaller inversion in grey overlapping with LGC6.1). PCA based on markers located in: (a1) the region spanned by the first part (from 0 to 8.7 cM) of LG6, spanned by LGC6.1; (a2) the region of LGC6.2, which overlaps with the LGC6.1 inversion; and (a3) in the collinear region. Unlike the first part, where three main groups were observed as in most of the other LGs, six groups were observed in the region where the two inversions overlap (from 8.7 to 29.3 cM), consistent with three homokaryotypes (blue, pink and red) and three heterokaryotypes (green, black and cyan). (b) Boxplot of observed heterozygosity for the six groups, comparing putatively inverted and collinear regions. (c) Distribution of the six groups across the transect (distance from the Crab end is shown). (d) Boxplots of nucleotide diversity (pi) for within (Inv.) and outside (Col.) the inverted region, in pairwise comparisons between the groups G1, G2 and G6 (homokaryotypes). (e) Pairwise comparisons of boxplots of divergence (*d_XY_*) between the same groups outside and inside the inverted region (E1)

The PCAs for most LD clusters revealed that individuals were aggregated into three genotypic groups (mainly on the first component) with intermediate genotypes between them absent or rare (Figures 4 and 5, Supporting Information Figure S1). This pattern suggests the presence of the two alternative homokaryotypes for a given rearrangement, with the heterokaryotypes in the middle, without recombination between the three genotypic groups. The rare exceptions (LGC4.1, LGC6.1, LGC7.2 and LGC12.2; Figure 5 and Supporting Information Figure S1) comprised some individuals with intermediate positions between homo‐ and heterokaryotypes, compatible with gene conversion or double crossovers that are known to occur in inversion heterokaryotypes (Stevison et al., 2011). Additionally, six groups of genotypes were observed in the region spanned by two LGCs (6.2 and 14.2), compatible with the presence of three homokaryotypes and three heterokaryotypes without obvious intermediate genotypes (Figure 5, Supporting Information Figure S1). This pattern is expected in regions of overlap between inversion events and so corroborates our interpretation of overlapping rearrangements on these two LGs.

In contrast, the collinear regions of the LGs containing LD clusters did not reveal obvious genotypic groups (Supporting Information Figure S1, right panels). Two exceptions were LG10, where three distinct groups were observed but the PC1 explained only 6.6% of variance; and LG12 where the number of groups and their limits are not very clear, with the PC1 explaining also a low proportion of variance (9.1%) when compared to all candidate inversions. An inversion may be present in one or the other LG, containing a small number of markers and/or composed by SNPs with a lower median intracluster LD (*r* ^2^ < 0.3), or the inversions detected on these LGs could extend further than our current estimates so that some SNPs currently included in the collinear regions are actually in the inversions. Alternatively, the grouping pattern could result from the influence of selection on markers that are not contained in a chromosomal rearrangement.

The sizes of the LD clusters in terms of map distance in the Crab linkage map (Table 1) were often large (11 LD clusters >5 cM) but were not significantly correlated with either the numbers of SNPs (*r* = −0.31) or the numbers of contigs (*r* = 0.40) that they contained. This was presumably because the parental individuals used in generating the Crab map varied in karyotype such that recombination was suppressed in one or both parents for some inversions (Figure 2c), reducing the apparent map length (which is based on the average of the maps from the two Crab family parents; Westram et al., 2018).

### 3.2 Genetic diversity

We compared heterozygosity (*H*_obs_) between the PCA groups in order to confirm the expectation based on the interpretation of LD clusters as inversions. Putative heterokaryotypes (central PCA groups) were expected to have higher heterozygosity than putative homokaryotypes. Heterokaryotypes were also expected to have higher heterozygosity than is observed in corresponding collinear regions. In most LGs, *H*_obs_ within the inverted regions of the putative heterokaryotypes was significantly higher than within the inverted regions of the putative homokaryotypes (Figures 4 and 5, Supporting Information Figures S2 and S3, Table S1). The only exception was observed for the putative inversions located in LG12, where one of the homokaryotypes did not show significant differences from the heterokaryotypes (Table S1). When comparing the putative heterokaryotypes with collinear regions for the same individuals, significantly higher *H*_obs_ was again revealed for almost all LGs containing LD clusters (Figures 4 and 5, Supporting Information Figures S2 and S3, Table S1). The only two exceptions were observed in LGC6.2 and LGC14.2 where at least one of the three heterokaryotypes had higher *H*_obs_ than in the collinear region but the differences were not significant after sequential Bonferroni correction. Overall, these results are consistent with expectations on the hypothesis that LD clusters represent inversions with the central PCA groups corresponding to the heterokaryotypes.

The comparison within the same LGs revealed that *π* was significantly different between the two homokaryotypes for different arrangements in 16 out of 21 tests after Bonferroni correction (17 LGCs plus additional comparisons for those with three arrangements present; Figures 4 and 5, Supporting Information Figures S4 and S5, Table S2). The arrangement with lower *π* can be inferred to be the derived arrangement, although the arrangements may also have been differently influenced by selection. The same tests performed in the noninverted regions of the same LGs (as control, using groups of individuals defined by their inversion karyotypes) revealed no significant differences with only one exception (LG12.1).

In all LGCs, divergence between arrangements, mean *d_XY_*, was higher than control values from collinear regions (which should equal the diversity, *π*, in those regions), implying accumulation of genetic differences since the origin of the inversion (Figures 4 and 5, Supporting Information Figures S6 and S7, Table S3). Although only eight cases remained significant after the Bonferroni correction, the sample size in one of the two groups was often small, resulting in low power.

### 3.3 Recombination patterns

Based on the hypothesis that LD clusters represent inversions, we predicted that recombination in heterokaryotypes would be absent or rare (and only due to gene conversion or double crossover) within the region covered by the LD cluster. For homokaryotypes with the alternative arrangement (relative to the Crab map reference) recombination patterns were predicted to be more consistent with the reversed gene order (Figure 2).

The identification of recombination events within four Wave families revealed no recombination event within the candidate inversions in 124 possible cases (parent–offspring combinations for each candidate inversion) in offspring genotyped from parents that were heterozygous for the different arrangements (karyotype “RA,” where “R” is the reference and “A” the alternative arrangement) (Table S4). In contrast, 145 recombination events were detected in the same regions of 1,137 cases in offspring from parents that were homozygotes for the inversions (RR and AA) (Table S4). Thus, recombination was suppressed in heterokaryotypes, as expected. A similar situation was observed in the Crab linkage map where high densities of SNPs were placed in small regions (corresponding to some of the LD clusters identified here) with absent or rare recombination events for parents that were inferred to be heterozygous for these inversions (RA) (Figure 2, Table 2). Among the 600 cases in offspring–parent pairs where the parent was homozygous for the reference arrangement (RR, as in the Crab map), 78 recombination events were detected within the region encompassed by the candidate inversions. With more informative markers available, 173 events were detected in the collinear regions of these same offspring–parent pairs (Table 2, Table S4). Thus, recombination was suppressed only in heterokaryotypes, as expected. Finally, 67 recombination events were observed within the regions encompassed by the candidate inversions among the 537 cases in offspring–parent pairs where the parent was inferred to be homozygous for the alternative arrangement (AA), while 117 recombination events were observed in the collinear regions for these same pairs (Table S4).

**Table 2:** Inferred inversion genotypes for the Crab and Wave parents for all linkage disequilibrium clusters (LGC), prediction of recombination suppression (A) in either Crab or Wave parents and of recombination patterns in Wave parents (B), as well as empirical support for those predictions

	CRAB mother	CRAB father	WAVE mother1	WAVE mother2	WAVE mother3	WAVE father1	WAVE father2	A‐Rec. suppression	B‐Rec. pattern	Data support
LGC1.1	RA^a^	RA	RR	RA	RR	RR	RR	Yes	Not informative^c^	A‐Yes; B‐NA
LGC1.2	RR	RR	RR	RA	RR	RR	RR	Yes	R‐like	A‐Yes; B‐Yes
LGC2.1	RR	RR	AA	RA	AA	AA	AA	Yes	A reversed from R	A‐Yes; B‐Yes
LGC4.1	RA	RA	RR	RR	RR	RR	RR	Yes	Not informative^c^	A‐Yes; B‐NA
LGC6.1	RR	RR	AA	AA	AA	AA	AA	‐	A reversed from R	A‐NA; B‐Yes
LGC6.2	RR	RR	A1A2	A1A1	A1A1	A1A1	A1A2	Yes	Not informative between A1 and A2	A‐Yes; B‐NA
LGC7.1	RR	RA	RR	RR	RR	RR	RR	Yes	R‐like	A‐Yes^b^; B‐NA
LGC7.2	NA	NA	NA	AA (or RR)	AA (or RR)	NA	NA	‐	‐	NA
LGC9.1	RR	RR	RR	RR	RR	AA	RR	‐	A reversed from R	A‐NA; B‐Partially^e^
LGC10.1	RA	RR	RR	RR	RR	RR	RR	Yes	R‐like	A‐Yes^b^; B‐Yes
LGC11.1	RA	RA	RR	RR	RR	RR	RR	Yes	Not informative^c^	A‐Yes^b^; B‐NA
LGC12.1	RR	RR	RR	RR	RA	RR	RR	Yes	R‐like	A‐Yes; B‐Yes
LGC12.2	RA	AA	NA	AA	AA	AA	AA	Yes	A‐like	A‐Yes^b^; B‐Yes
LGC14.1	RR	RA	AA	AA	AA	AA	AA	Yes	A reversed from R	A‐Yes^b^;B‐Largely^d^
LGC14.2	RR	RA1	A1A1	A1A1	A1A1	A1A1	A1A1	Yes	Not informative between A1 and A2	A‐Yes^b^; B‐NA^d^
LGC14.3	NA	NA	NA	NA	NA	NA	NA	–	–	NA
LGC17.1	RR	RR	RA	RA	RA	AA	RA	Yes	A reversed from R	A‐Yes; B‐NA

a R—reference, defined as the most common arrangement in the Crab transect end; A—alternative; A1 and A2 are two different alternative arrangements. NA—not available.

b Information gathered from the Crab map (Westram et al., 2018).

c Both Crab parents were heterozygotes for the inversion.

d One exception contradicting the expected pattern out of 28.

e RR supported, recombination in AA not informative.

Since single crossovers are expected to be much more frequent than double crossovers or gene conversion, we classified the offspring haplotypes as more consistent with either the most frequent Crab gene order (R) or a gene order (A) that is reversed in the putatively inverted region, or as uninformative if they were equally consistent with both gene orders. All the informative haplotypes except one (99/100) were more consistent with the parent's karyotype (inferred from the PCA) than with the other gene order. Among these, 38 out of the 39 informative events were cases where the parent had the alternative gene order (AA) rather than with the order inferred for the Crab linkage map (RR). Altogether, recombination patterns compatible with the presence of inversions were observed for all LGCs except three, where difficulties in inferring the parent genotypes from the SNPs available (LGC7.2 and LGC14.3), as well as lack of informative events (LGC9.1), precluded inferences based on the observed recombination patterns (Table 2). The inversion status of LGC14.3 also has only weak support in terms of variance explained by PC1. Not all of the remaining inversions received equal support. While 13 inversions were supported by recombination suppression in heterokaryotypic parents, recombination consistent with an inverted map between the reference and alternative arrangements was only available for three inversions (LGC2.1, LGC6.1 and LGC14.1), since the parents’ genotypes for the remaining inversions were not informative (Table 2).

All recombination patterns were consistent with existing linkage group assignments, with no concentration of inferred recombination events at the boundaries of LGCs that could not be resolved by a change in gene order. This rules out the possibility that the rearrangements underlying LGCs were translocations.

### 3.4 Inversion frequencies along the transect

The distributions of the different alternative arrangements for each LG cluster across the transect were highly variable. Some LGCs had homokaryotypes that were present only or mainly in one of the ecotype‐specific habitats (e.g., LGC6.1) while others had one homokaryotype that was found mainly in the central part of the transect (e.g., LGC11.1) or both homokaryotypes present across the entire transect (e.g., homokaryotypes of LGC1.1) (Figures 4 and 5, Supporting Information Figure S1).

**Figure 6:**
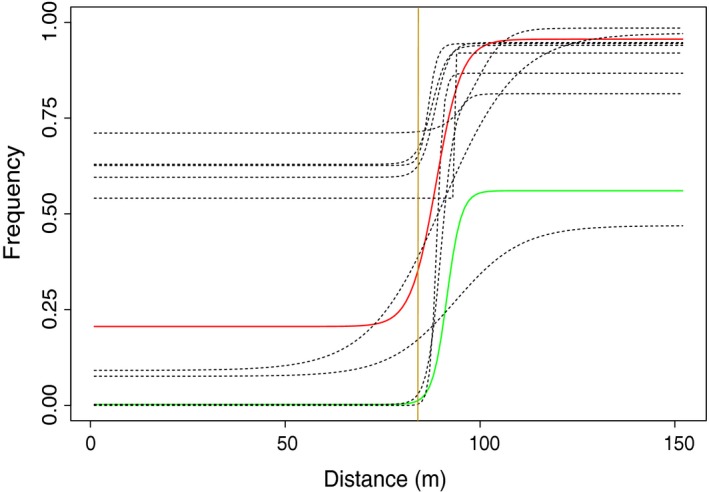
Inversion frequencies across the transect. Predicted frequencies (dashed lines) for the 11 inversions that fitted a clinal model (∆AIC > 10) across the sampled transect (*x*‐axis). The clines for two inversions that contain many outlier SNPs (Westram et al., 2018) are highlighted: LGC17.1 (green) and LGC6.1 (red). The main habitat transition is shown (orange vertical line)

Most inversions showed a significant clinal change in arrangement frequency across the transect (except LGC1.2, LGC6.2‐group 2, LGC7.2; Table S5). The asymmetrical cline model was never a better fit than the symmetrical cline (not shown). Additionally, the cline was a poor fit to the data (deviance explained <10%), despite meeting the ∆AIC criterion compared to a constant frequency, for three other inversions (LGC9.1, LGC11.1 and LGC14.2‐group 2), which were excluded from subsequent analyses. Both the widths and the centres of the clines varied significantly (*p = *1.854E‐13 and 1.284E‐35, respectively) among inversions (Figure 6): the estimated centres varied between 84 and 99 m for LGC12.1 and LGC10.1, respectively, and the estimated widths varied between ~1 and 36.6 m for LGC12.1 and both LGC14.3 and LGC14.1, respectively. For one inversion (LGC14.2‐group 4), a cline with one of the arrangements fixed in both transect ends was as good a fit as a cline with unconstrained end frequencies, suggesting strong divergent selection. For seven other inversions (LGC6.1‐group 4 Crab, LGC7.1 Wave, LGC10.1 Wave, LGC12.2 Wave, LGC14.1 Wave, LGC14.2‐group 1 Wave, LGC17.1 Crab), a cline with one arrangement fixed in one of the transect ends (two in Crab and five in Wave) was as likely as a fit with unconstrained frequencies; whereas the remaining inversions were all polymorphic in both transect ends (Supporting Information Table S5, Figure 6).

## 4 DISCUSSION

Early work emphasized the impact of chromosomal inversions on adaptation and speciation (Dobzhansky, 1970; Sturtevant, 1926, 1938) but, subsequently, structural rearrangements received less attention, despite some prominent exceptions (e.g., Balanyà, Huey, Gilchrist, & Serra, 2009; Coluzzi, Sabatini, della Torre, Di Deco, & Petrarca, 2002). More recently, inversions have been detected in many systems (Wellenreuther & Bernatchez, 2018), prompting renewed interest in the role they play in local adaptation and speciation. Advances in sequencing technologies and genomics have promised to make structural variation more readily detectable (Alkan, Coe, & Eichler, 2011). However, this task remains difficult for many nonmodel organisms, with approaches based on short reads especially prone to both high rates of false positives and negatives (Sedlazeck et al., 2018; Lledó & Cáceres, 2013 and refs. therein). In practice, the presence of inversions has often been inferred from patterns of divergence in genome scans (e.g., Jones et al., 2012) with subsequent confirmation using sequencing or genetic mapping approaches (e.g., Twyford & Friedman, 2015). This can give a biased impression of the role of inversions because suppression of recombination makes their contribution to adaptive differentiation easier to detect than the contribution of loci in freely recombining regions and because inversions that are not differentiated between populations may remain undetected.

The linkage‐disequilibrium‐based approach we implemented here, using data gathered from a hybrid zone between two *Littorina saxatilis* ecotypes, allowed us not only to detect rearrangements de novo but also to infer their frequencies and putative contribution to ecotype divergence. This not only circumvented the need for subsequent genotyping to estimate karyotype frequencies but also contributed to reducing the number of candidate rearrangements for further validation because attention could be focused on those with strong clinal patterns. Providing that linkage maps (recombination information) or high‐quality reference genomes are available, the candidate rearrangements detected by their LD signatures can be confirmed and their type (e.g., inversions or translocations) can also be identified. Detection of rearrangements using information from LD between markers, complemented by PCA and genetic diversity information, was proposed previously (Kemppainen et al., 2015). Our results further demonstrate the utility of this approach. However, clusters of markers in high LD can be generated by other processes, especially when selection acts in opposition to gene flow on regions of low recombination (Burri, 2017), and the choice of thresholds in LDna has not been validated by comparison to simulations. This means that the LD clusters themselves are only indicative. Observing distinct genotypic clusters through PCA, with the expected patterns of heterozygosity, supports the hypothesis that LD clusters represent inversions. However, additional independent lines of evidence are needed to confirm the chromosomal rearrangements. This evidence can come from recombination mapping, as we used here.

We detected 17 candidate rearrangements, including three that correspond to LD blocks reported by Westram et al. (2018). This number is dependent on the parameter values chosen in the initial LDna and PCA and may be an underestimate because we aimed to set conservative thresholds. All candidates, except three (LGC7.2, LGC9.1 and LGC14.3), were supported by recombination patterns from Crab or Wave families, which tends to confirm that our criteria were stringent. Due to the limited number of offspring available to identify recombination events and the particular genetic composition of the parents used in the crosses, not all of the remaining inversions were equally supported. Thus, future validation of some of these candidates using cytogenetics (as in Lee et al., 2017) and/or long‐read sequencing is desirable.

Studying other localities across the wide geographic and environmental range occupied by this species may well reveal further rearrangements. We believe that this approach can be extended successfully to other case studies with similar data available, but it is likely that thresholds (e.g., minimum number of loci within an LD cluster) will need to be fine‐tuned through exploratory analyses in order to make informed decisions concerning some parameters.

The number of inversions detected in *L. saxatilis* is high when compared with other systems (Wellenreuther & Bernatchez, 2018), likely at least partly due to the use of different methodology. If inversions cause a fitness cost on heterokaryotype individuals due to the generation of unbalanced gametes when single crossovers occur within inversions, then this large number of polymorphic inversions could represent a substantial load. However, although it occurs in plants, this type of cost seems rare in animals (Hoffmann & Rieseberg, 2008; Rieseberg, 2001). The range of inversion sizes in this system is within that observed for other species (Wellenreuther & Bernatchez, 2018). However, it is important to keep in mind that inversion sizes, defined according to the Crab map, are unlikely to correlate well with physical lengths because the parents used to construct that map were sometimes heterozygotes for those inversions (e.g., LGC1.1 and LGC4.1; Figure 2). Also, this approach alone cannot be used to infer rearrangement breakpoints with precision. The coordinate ends we present must be interpreted as boundaries of the regions influenced by the rearrangements and some of the SNPs at the ends of an LD cluster may actually be outside, although close to the rearrangements’ breakpoints. Nevertheless, assessment of the genotypic information from the Wave families allowed us to verify that the inferred boundaries were compatible with inversions (changes in orientation within the same chromosome) rather than with the exchange of genetic material between chromosomes through translocations.

The levels of observed heterozygosity further supported the inversion status of the LD clusters. The middle groups identified in the PCA presented higher *H*_obs_ within each of the LD cluster regions than the other two groups, as expected for heterozygotes for the inversions relative to the homokaryotypes. For most LD clusters, the two homokaryotypes presented significant differences in nucleotide diversity. This imbalance is expected for inversions where the derived arrangement is young, having originated recently as a single haplotype. Over time, the younger haplotype is expected to accumulate diversity through mutation but an imbalance may remain because the less common haplotype has a smaller effective population size and because of the strong effect of background selection and selective sweeps on both haplotypes. Divergence between arrangements is also expected to accumulate with time, due to suppression of recombination in heterokaryotypes. This prediction is generally supported by our data in some LGCs (Supporting Information Figure S7 and Table S4). Observed divergence may also be influenced by selection and by gene flux due to double recombination and gene conversion. It would be premature to interpret these diversity and divergence data in terms of inversion ages, but they do suggest that the origins of the inversions pre‐date postglacial colonization of the Swedish coast (<10,000 generations ago).

Recombinants between the three or six genotypic groups from the hybrid zone were generally absent in the transect within rearranged regions (Figure S1). The rare exceptions could result from gene conversion or double crossovers, which are known to occur within inversions, although at low rates (~10^−4^ for double crossovers and ~10^−5^ for gene conversion in *Drosophila*; Stevison et al., 2011). Missing data at informative markers for distinguishing the inversion genotypes (defined according to the PC1 scores) could also result in apparent intermediacy of individuals in the PCA. A close inspection of their genotypes for informative markers supports both explanations (not shown). Nevertheless, the recombination information gathered from the Wave families showed recombination to be absent (or rare) within the candidate inverted regions for the heterokaryotype parents. This, together with recombination patterns, is consistent with a reversed gene order relative to the Crab map and provides independent support that these candidate regions correspond to inversions. According to our results, most LGs (at least 11 out of 17) carry inversions, together encompassing ~25% of the total number of SNPs analysed. Given the suppressed‐recombination effects observed here, these inversions are likely to play a major role in shaping the recombination landscape in this system.

Given that many inversions are segregating in this population, an important question is whether they contribute to local adaptation. Are these inversions influenced by divergent selection? Westram et al. (2018) estimated that the majority of outlier SNPs were clustered in regions that overlap with the inversions that we detected in LG6, 14 and 17. Our cline‐fitting analysis of most inversions revealed that their frequencies change clinally across the transect, with varying width and position. However, simulations of this system by Westram et al. (2018) show that a clinal pattern can appear for neutral loci due to isolation by distance and a genome‐wide barrier effect close to the habitat transition. Therefore, significant cline fits are not, in themselves, good evidence for divergent selection.

The majority of arrangements remain polymorphic at one or both of the transect ends: a pattern that is inconsistent with a simple model of direct divergent selection generating the steep clines in inversion frequencies that we observe. Given the estimated cline centres and widths, a gene flow–divergent selection balance alone predicts arrangement frequencies within 1% of fixation at the ends of our transect for all clinal inversions. This prediction is independent of the value of dispersal because the greater the dispersal, the stronger the selection that is required to explain the observed cline width. Most observed clines had at least one end frequency far from this expectation. Westram et al. (2018) found the same pattern for SNPs and considered several possible explanations: weak indirect divergent selection on neutral loci linked to selected loci, selection on polygenic traits or a combination of divergent and balancing selection that shapes the allele or arrangement frequencies, maintaining polymorphism in one or both habitat ends but with different equilibria. Observations from multiple systems have shown that inversions are often under the influence of balancing selection, which facilitates the retention of polymorphism for many generations and may explain why many observed polymorphic inversions are so old (Butlin 2005; Wellenreuther & Bernatchez, 2018 and refs therein). Dobzhansky (1950) demonstrated both heterosis and differences in equilibrium frequencies between localities for inversions in *D. pseudoobscura*. Consequently, we suggest that a combination of balancing and divergent selection (within and between ecotypes, respectively) is a plausible explanation for the inversion clines in *L. saxatilis*. Clearly, further simulations and empirical observations will be needed to test the hypothesis of balancing and divergent selection and exclude alternative explanations. Direct estimates of selection might be possible, for example, using field transplants, but for arrangements on LG6, 14 and 17 there is already good evidence for a component of divergent selection from the analyses of SNP clines in Westram et al. (2018). Balancing selection has been demonstrated for adaptive shell colour traits in this system (Johannesson & Butlin, 2017) and may also influence other traits. If this hypothesis is confirmed, further studies should also aim to distinguish among the different forms of balancing selection that may play a role (e.g., frequency‐dependent selection, heterosis or spatially variable selection) and, if heterosis is observed, to understand how it is generated (e.g., associative overdominance or coadaptation; Butlin & Day, 1985; Kirkpatrick, 2010). Further work is needed to test the hypothesis of a combination of balancing and divergent selection, seeking observations or experiments that clearly distinguish it from the other hypotheses mentioned above. Nevertheless, we suggest that this possibility should also be considered for inversion polymorphisms in other species.

Balancing and/or divergent selection between habitats could have maintained inversions for long periods of time, resulting in the high diversity and divergence for some inversions noted above (Faria, Johannesson, Butlin, & Westram, 2019) . These inversions may have been segregating in ancestral populations where analogous Crab and Wave environments occur, and subsequently underpinned rapid adaptation to the Crab and Wave habitats following colonization of the Swedish coast. The presence of many inversions encompassing a large proportion of the genome can explain why we observe such a high number of divergent loci between ecotypes after such a short time since postglacial colonization. In addition, more recent gene flow between populations is likely to contribute to the efficient spread of inversions, especially if they contain adaptive variation (Johannesson et al., 2010; Morjan & Rieseberg, 2004). In these ways, inversions could help to explain the pattern of sharing of loci putatively influenced by selection, which is greater on smaller than on large geographic scales (Westram et al., 2016). Thus, determining the ages and spatial distributions of the inversions described here will be critical to further understanding of local adaptation and the evolution of reproductive isolation between *L. saxatilis* ecotypes.

Finally, complementary evidence to understand the link between inversions, adaption and selection can come from determining the genes present within inversions and the phenotypes that are associated with inversion polymorphisms. Although most of the outlier SNPs identified by Westram et al. (2018) are located within inversions, it is unlikely that they are all under direct selection. Genome annotation for *L. saxatilis* (M. Panova and T. Larsson, personal communication) will allow the identification of candidate genes and functions that may play a role in adaptation and ecotype divergence. Association mapping in this hybrid zone has already revealed that a large proportion of the genetic variance observed for some key adaptive phenotypes may be explained by genetic variation within some of these inversions (Westram et al., 2018). Studies with additional localities and further phenotypes will extend this connection.

We have demonstrated the power of LD patterns to detect inversions. There may be some bias in this approach towards inversions associated with local adaptation. Nevertheless, in our study site, inversions apparently make a major contribution to adaptive divergence.

## AUTHOR CONTRIBUTIONS

R.F., R.K.B., K.J. and A.M.W. conceived the study; A.M.W., M.Rav, K.J., A.R.L., E.M.L., M.P. and R.K.B. collected data; R.F., A.M.W., P.C., T.L., H.M., M.Raf and R.K.B. analysed data; R.F. and R.K.B. wrote the first draft; and all authors contributed to subsequent versions of the manuscript.

## Data Availability

DNA sequence data and genotypes to assess recombination in the Wave family were archived in NCBI SRA (PRJNA493979) and Dryad (https://doi.org/10.5061/dryad.72cg113), respectively. The remaining data had been previously archived in Dryad and SRA (https://doi.org/10.5061/dryad.bp25b65 and https://www.ncbi.nlm.nih.gov/sra/SRP155664, respectively).
