# Supplementary figures and images for: Multiple chromosomal rearrangements in a hybrid zone between Littorina saxatilis ecotypes
Source: Mol Ecol. 2019 Feb 25;28(6):1375–93. doi: 10.1111/mec.14972 (PMC6518922; doi:10.1111/mec.14972)

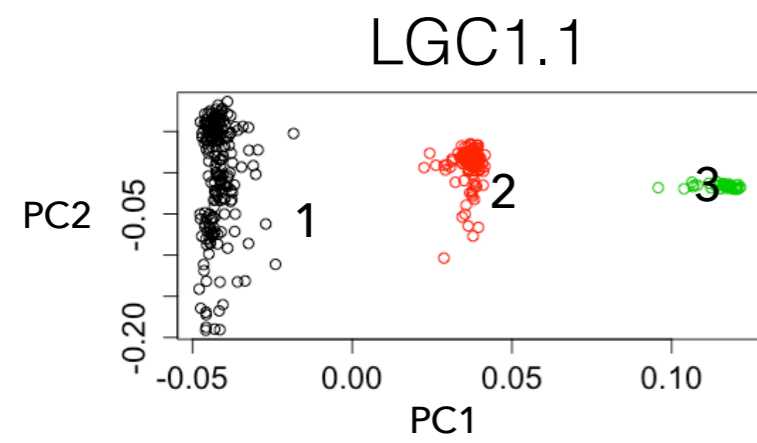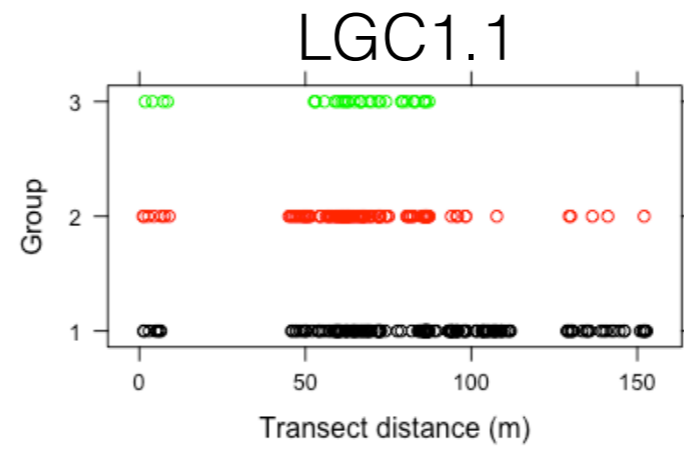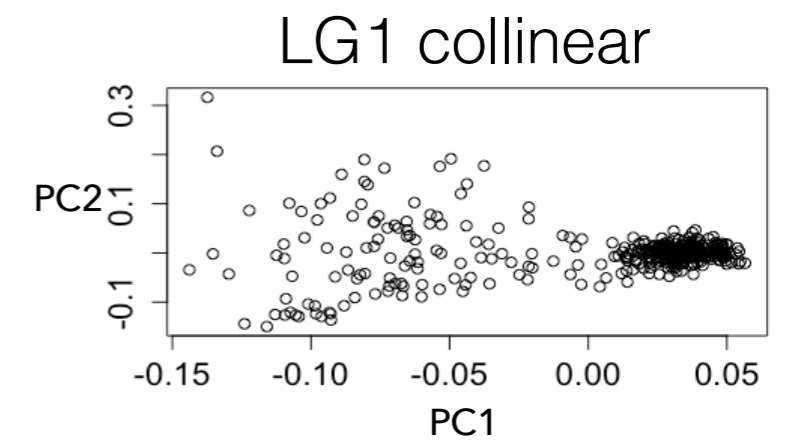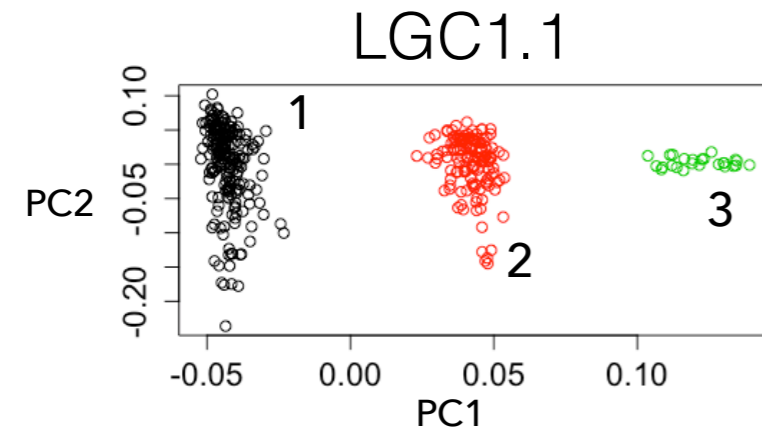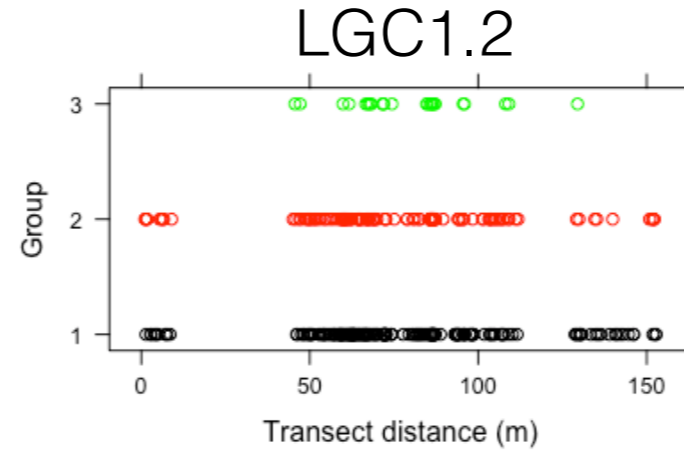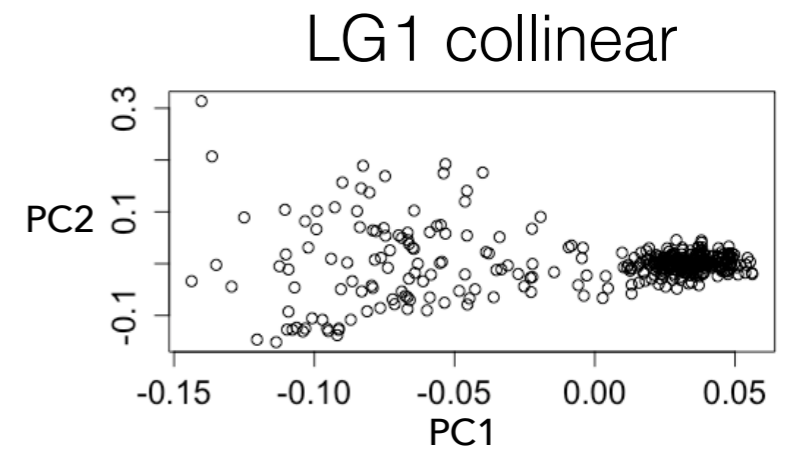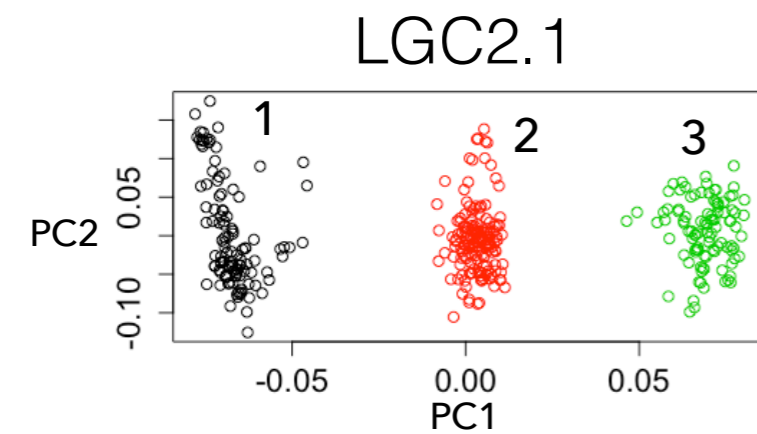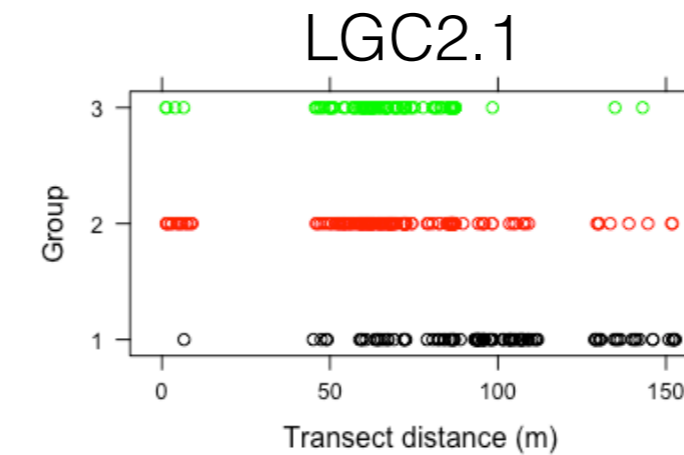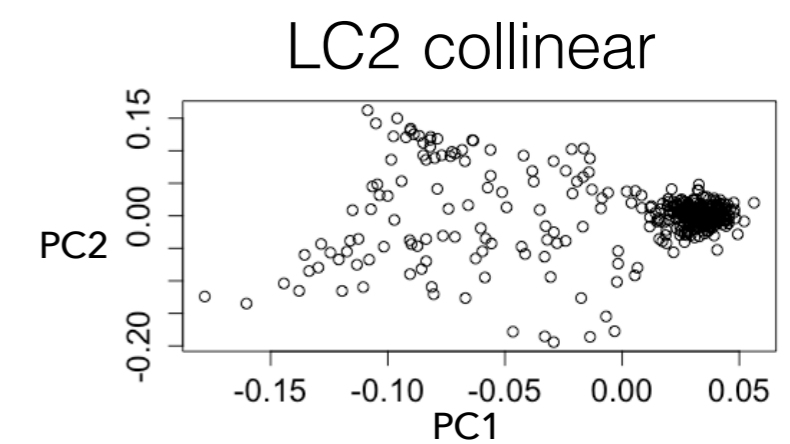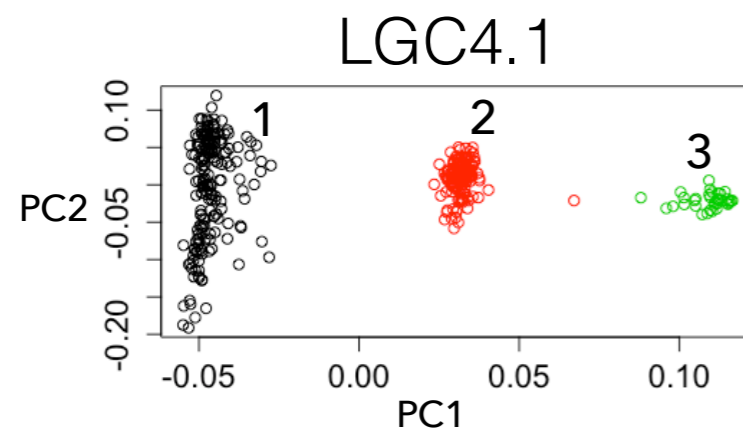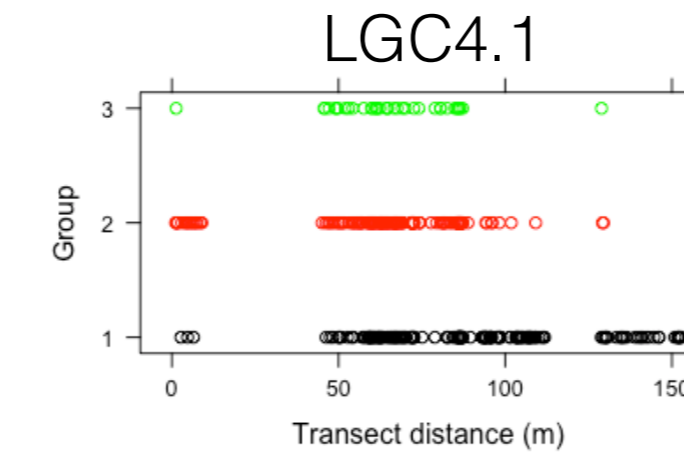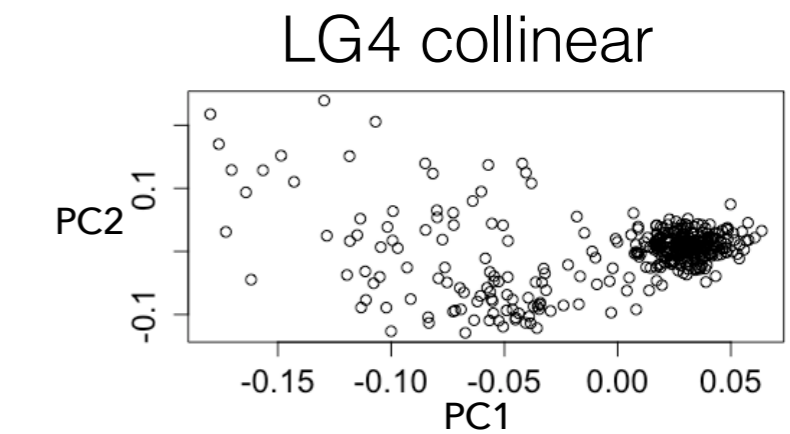

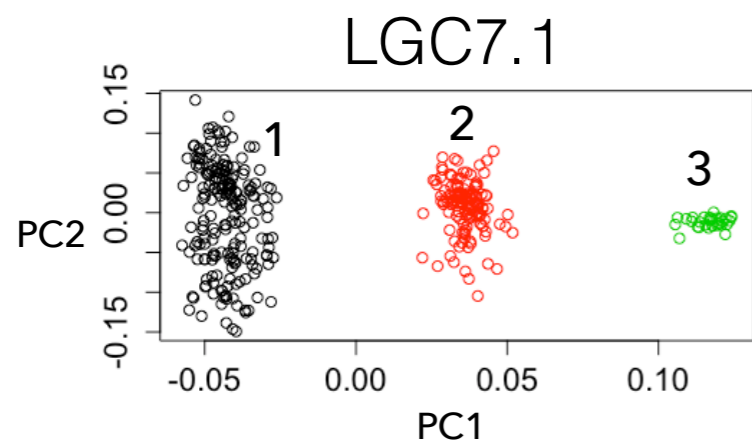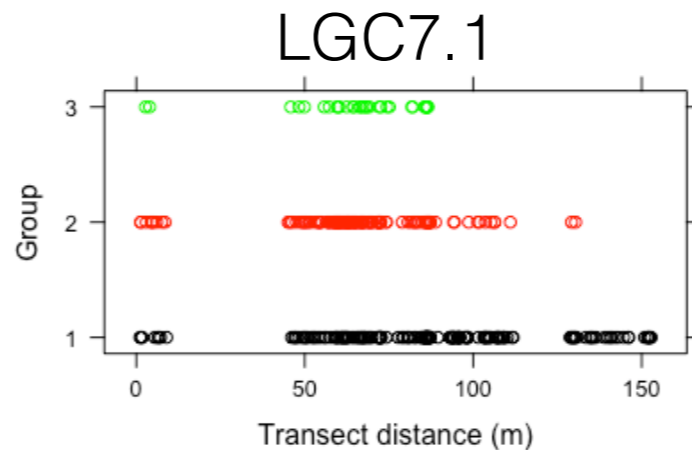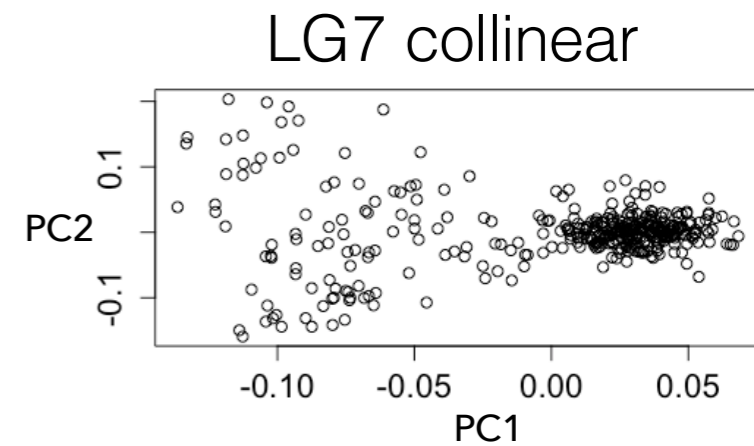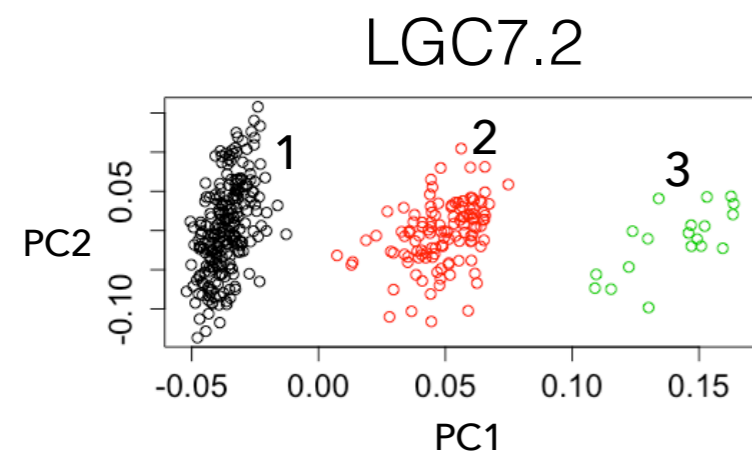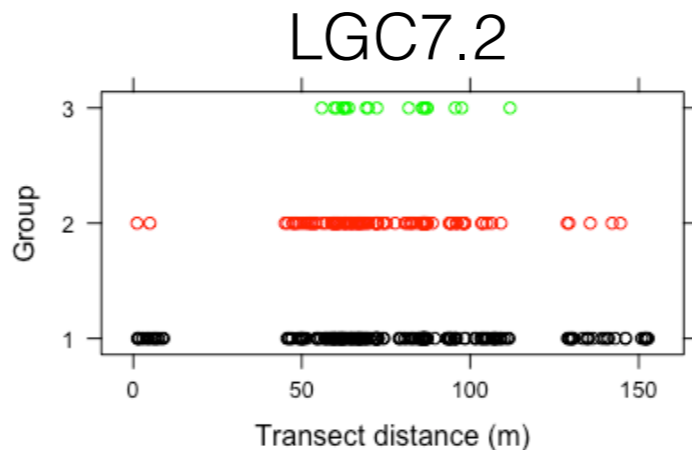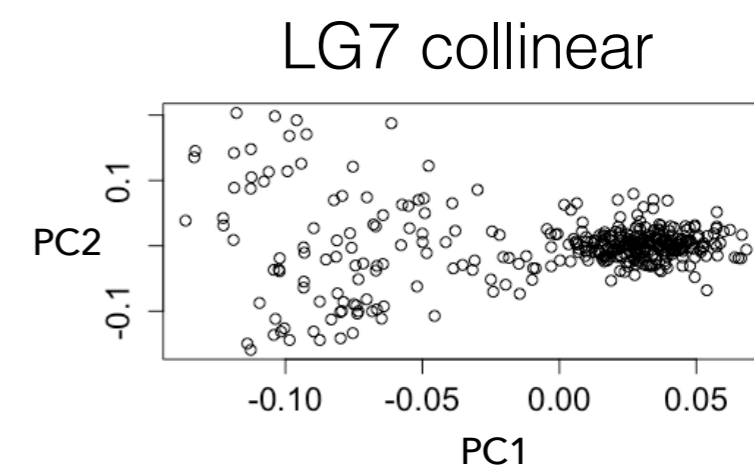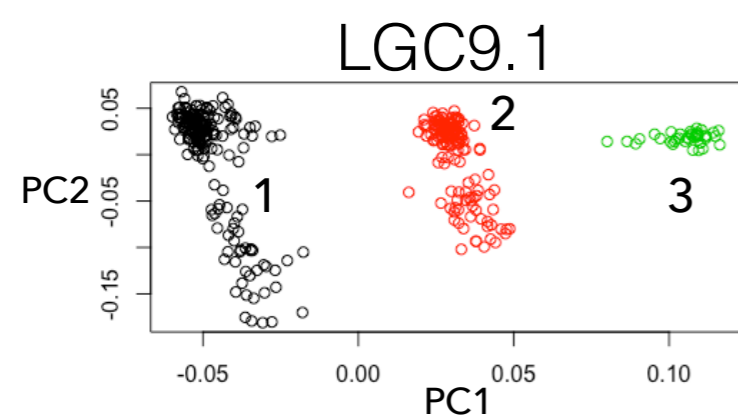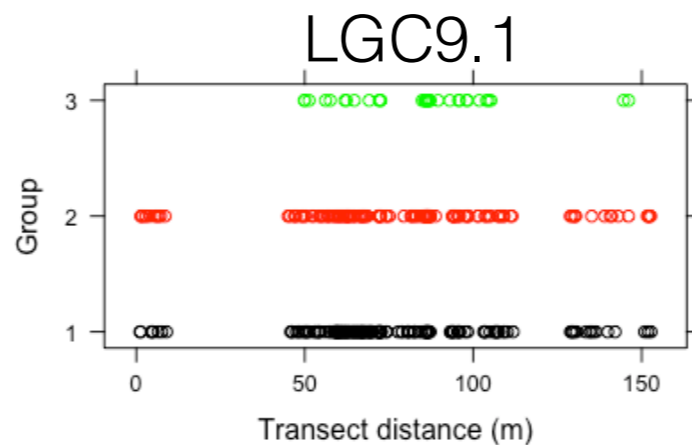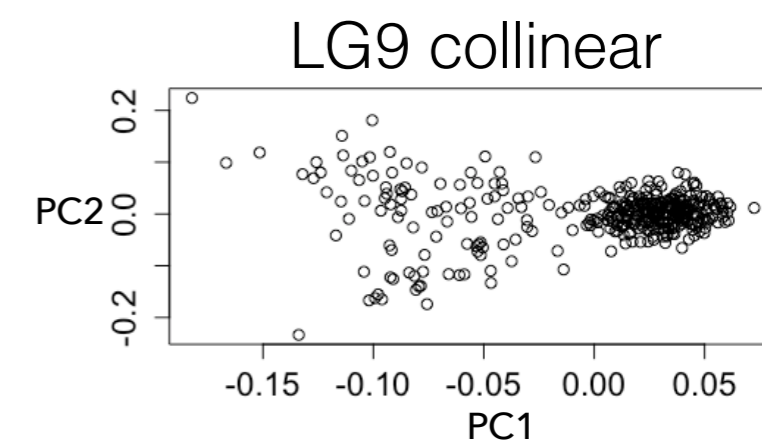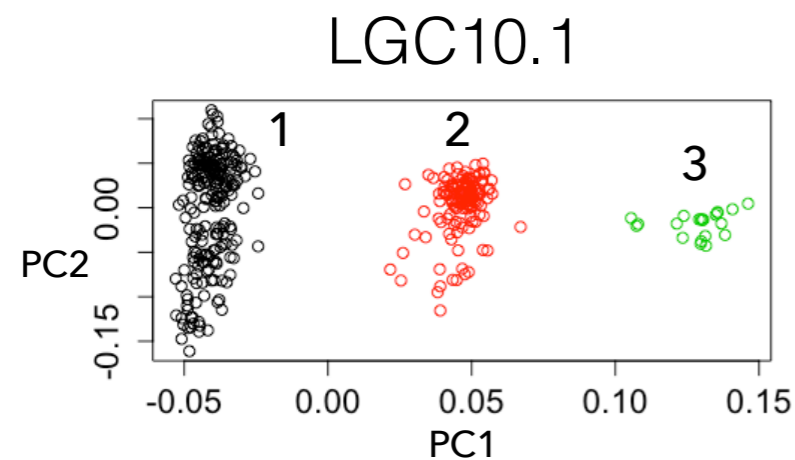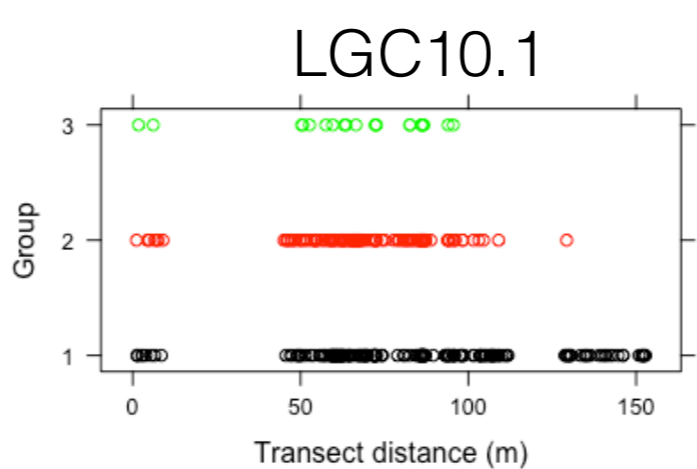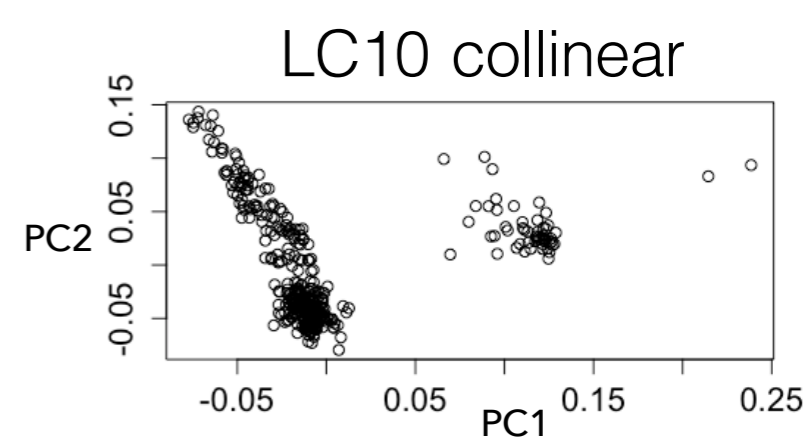

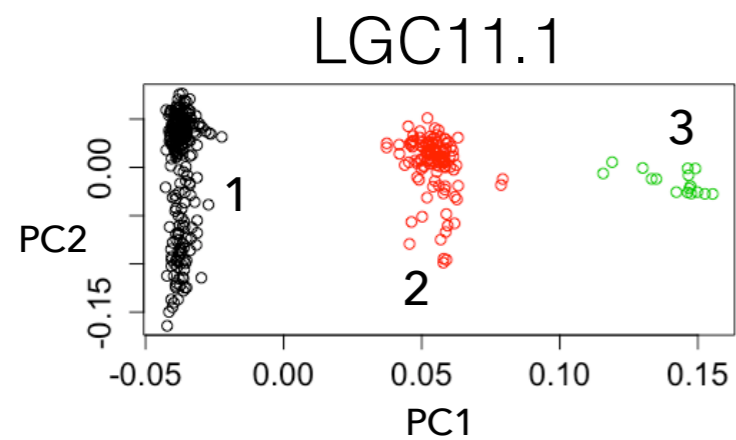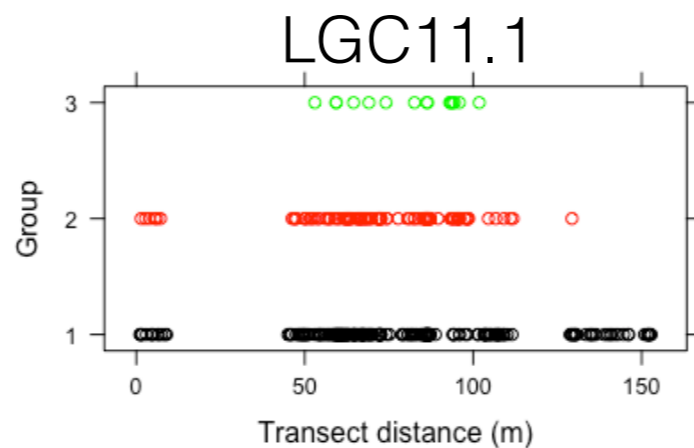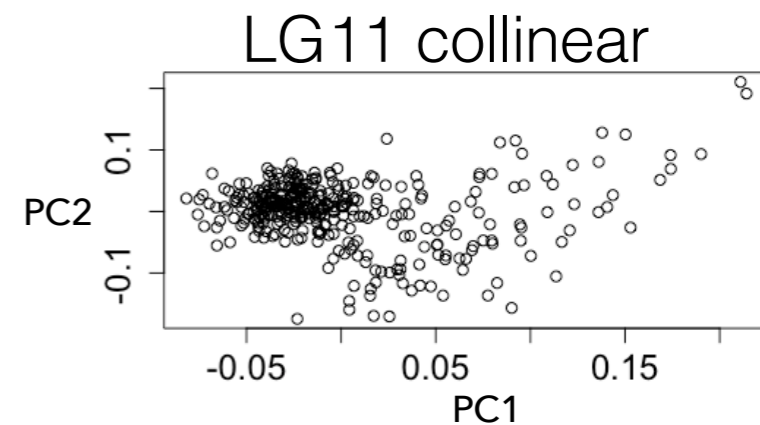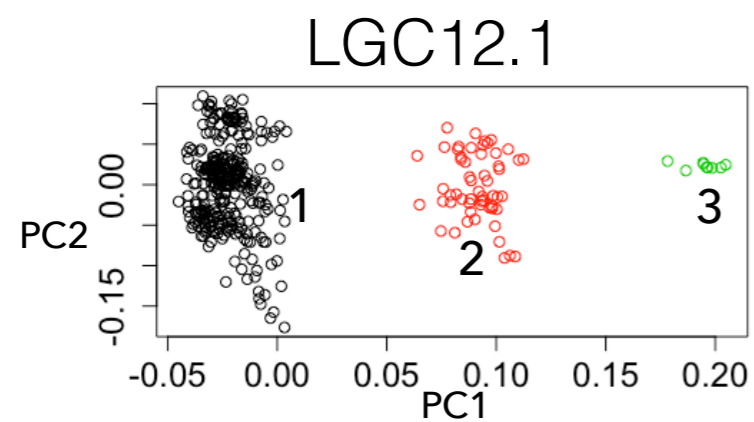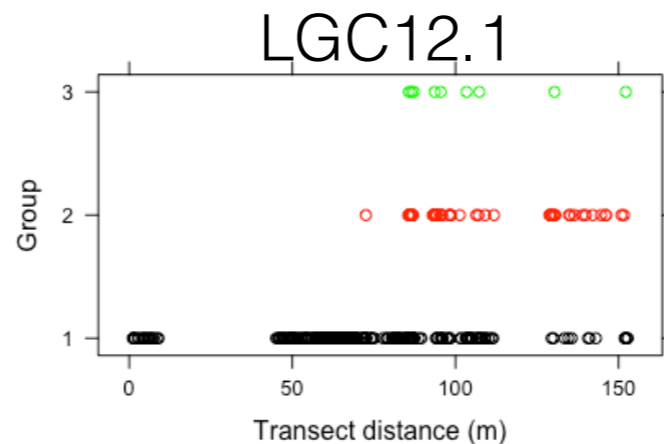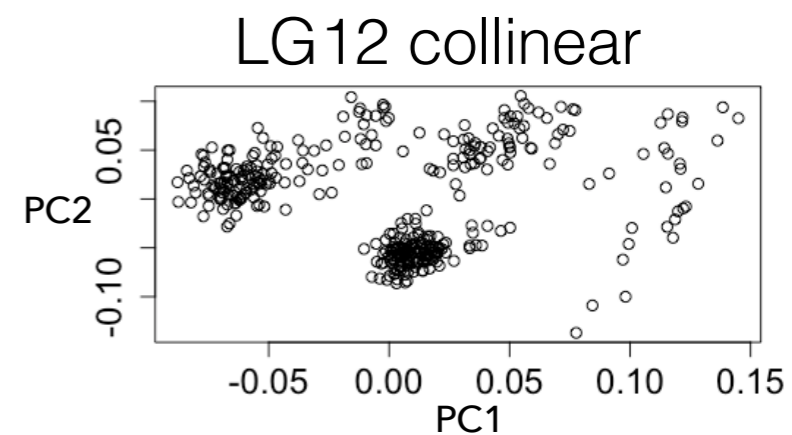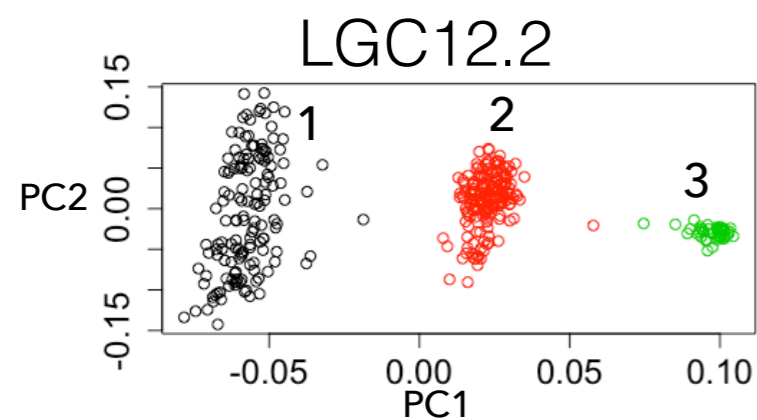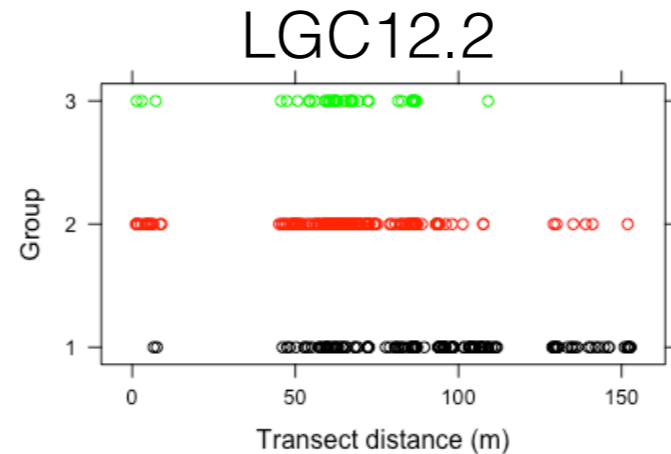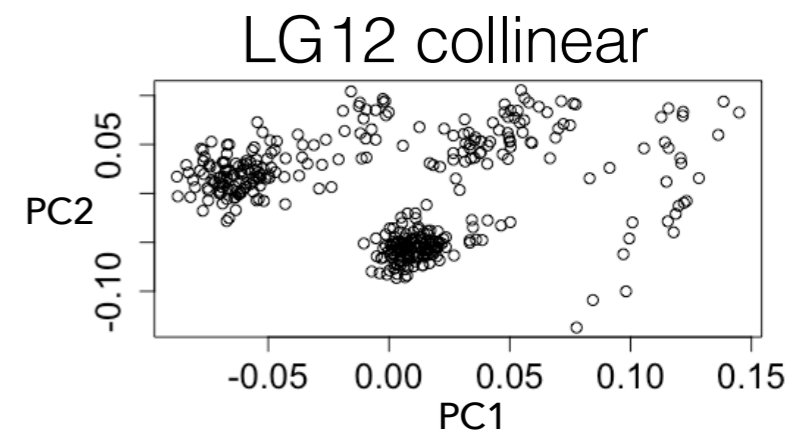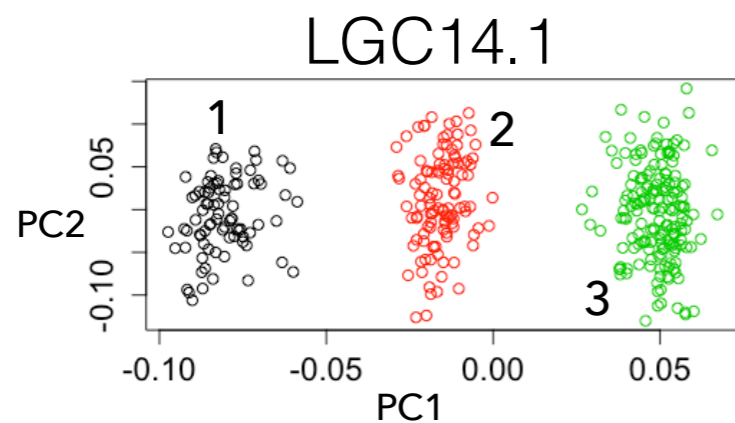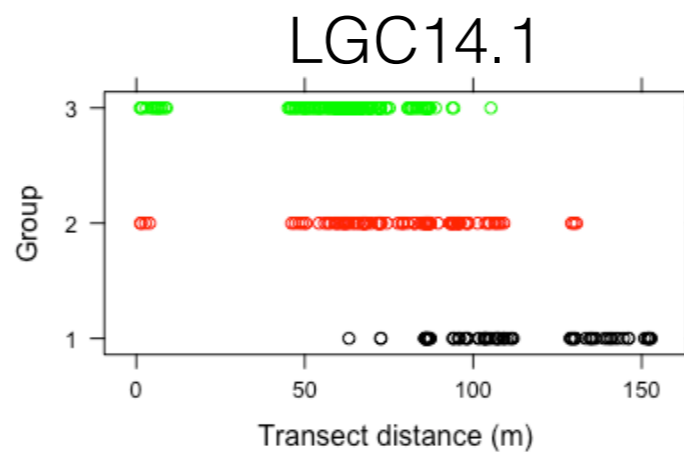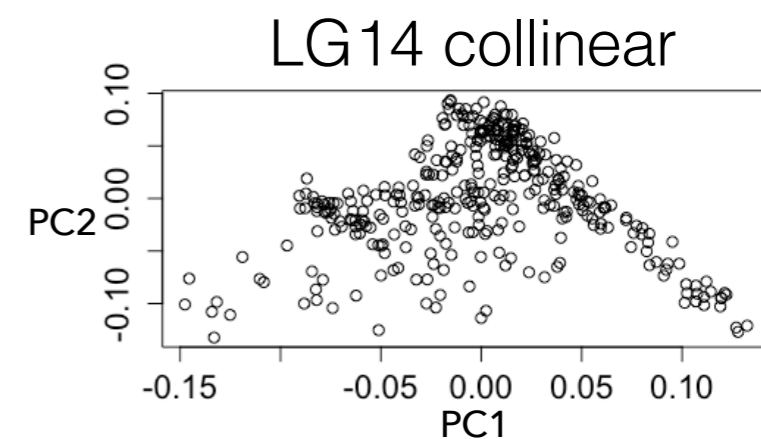

LGC14.2

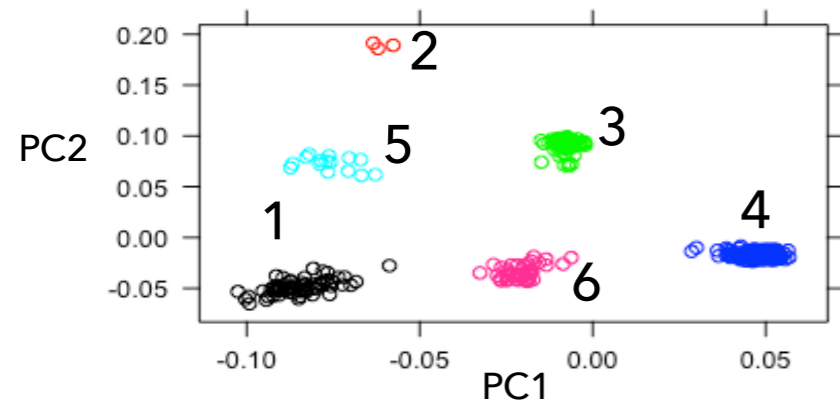

LGC14.2

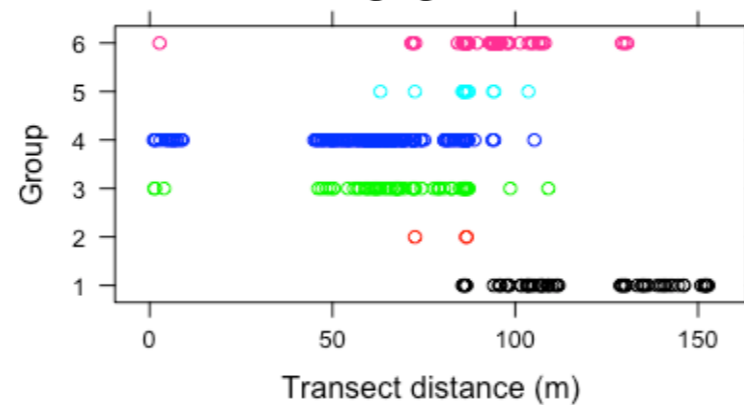

LG14 collinear

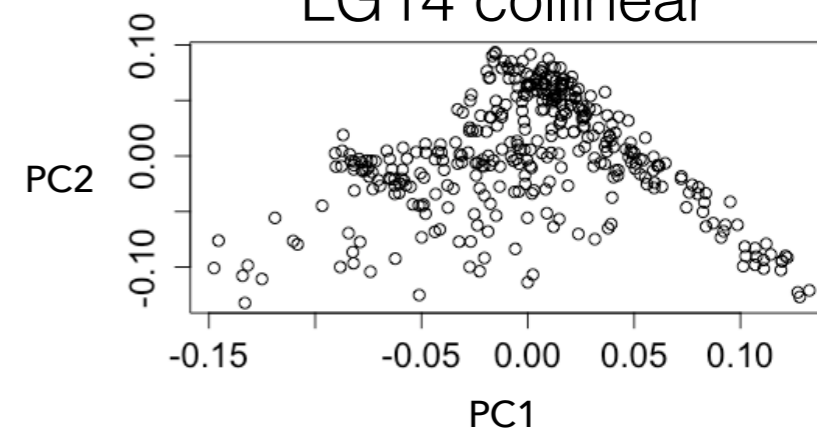

LGC14.3

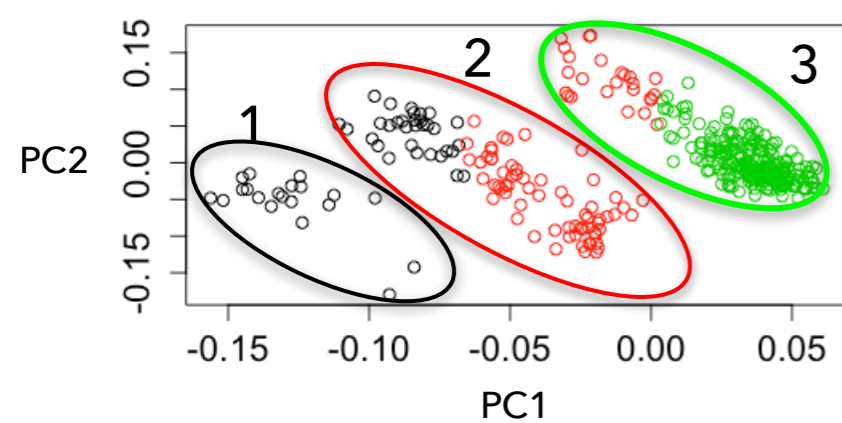

LGC14.3

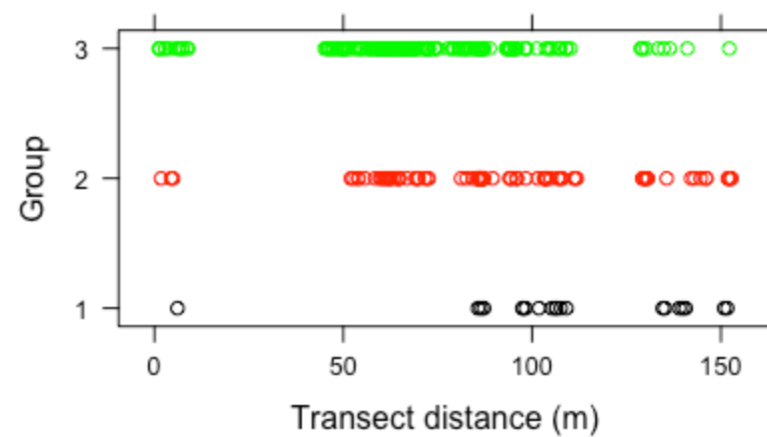

LG14 collinear

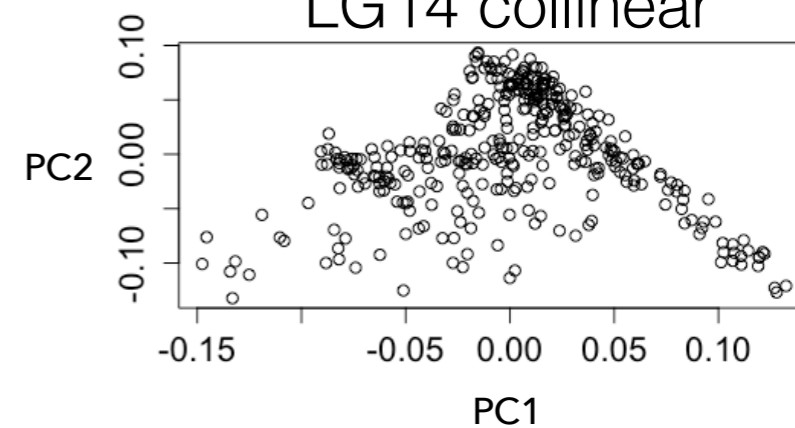

Supplement: Supplementary file 1 [file MEC-28-1375-s001.pdf]

LGC1.1

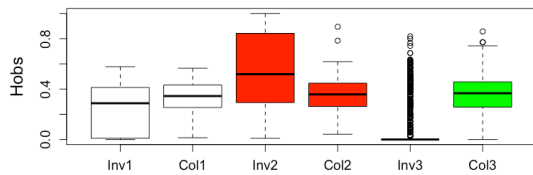

LGC1.2

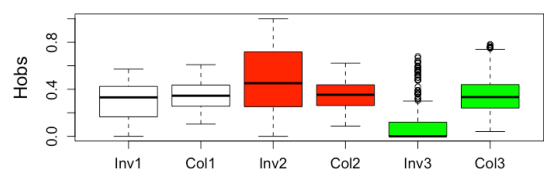

LGC2.1

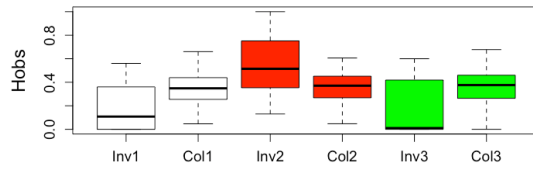

LGC4.1

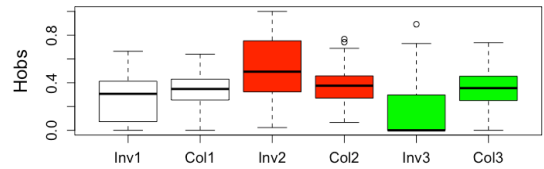

LGC6.1

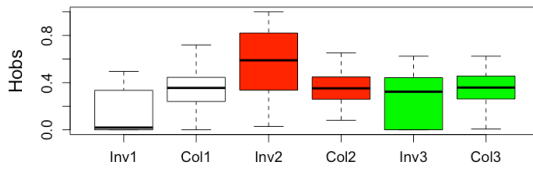

LGC7.1

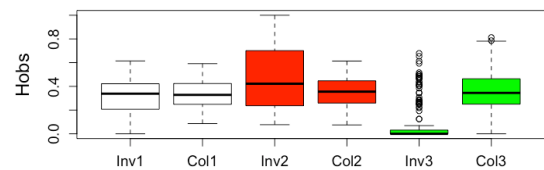

LGC7.2

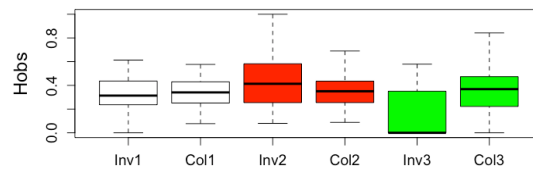

LGC9.1

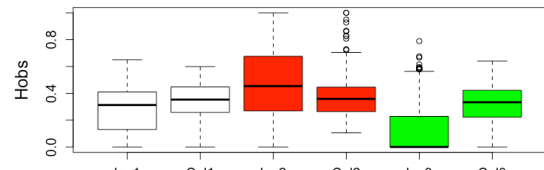

LGC10.1

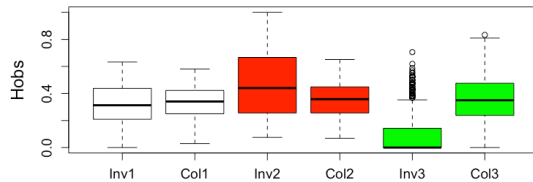

LGC11.1

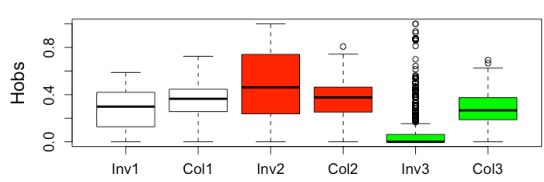

LGC12.1

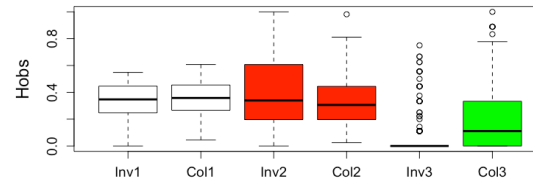

LGC12.2

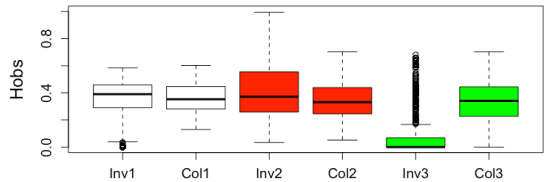

LGC14.1

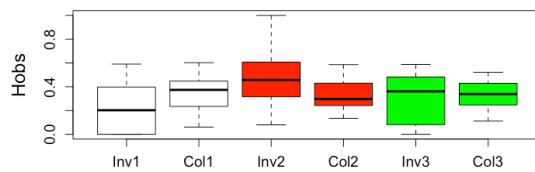

LGC14.2

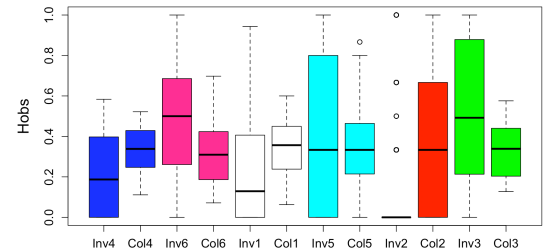

LGC14.3

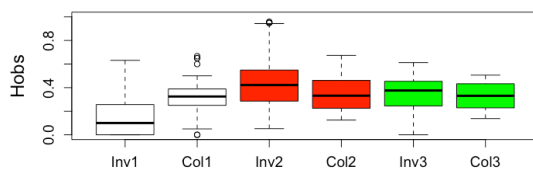

LGC17.1

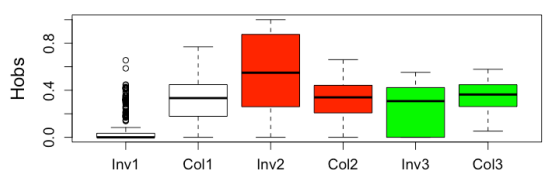

Supplement: Supplementary file 3 [file MEC-28-1375-s003.pdf]

**LGC1.1**

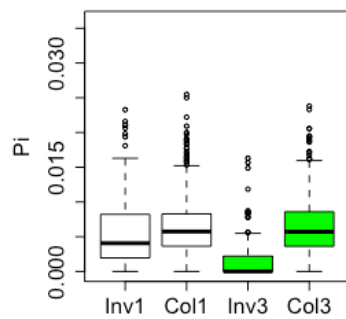

**LGC1.2**

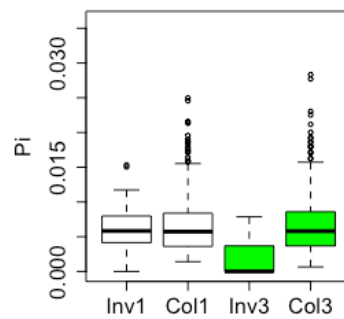

**LGC2.1**

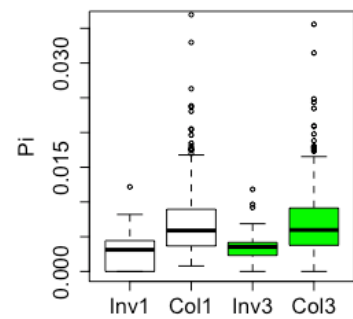

## LGC4.1

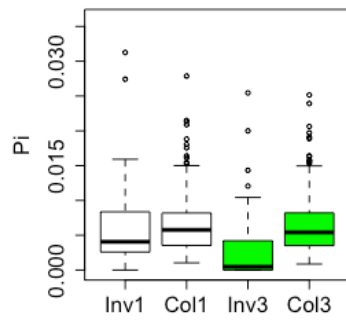

### LGC6.1

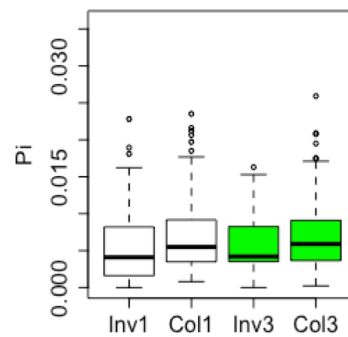

**LGC7.1**

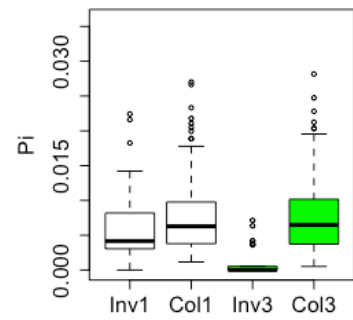

**LGC7.2**

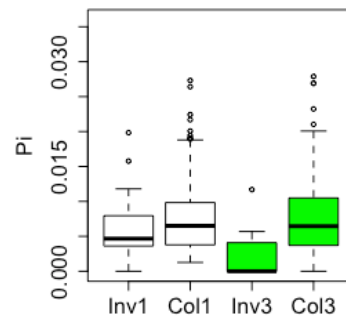

### LGC9.1

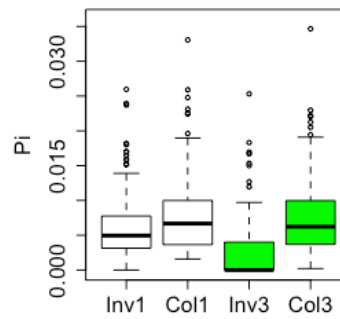

**LGC10.1**

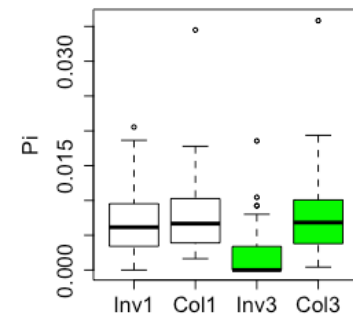

LGC11.1

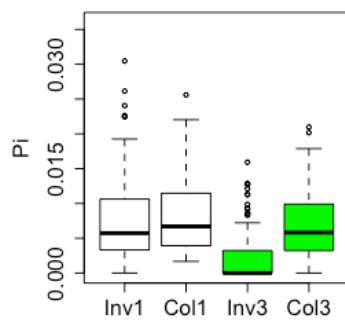

LGC12.1

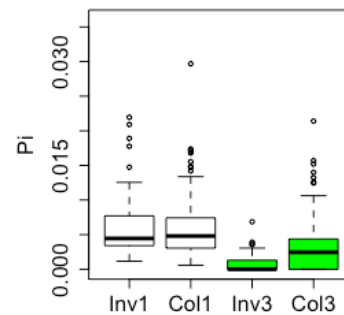

LGC12.2

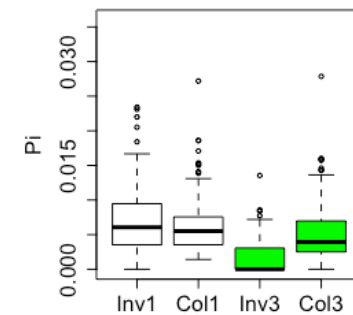

**LGC14.1**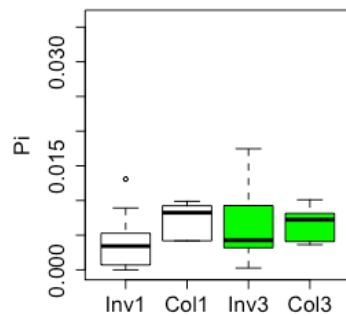**LGC14.2 1vs2**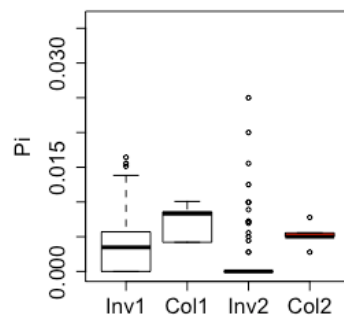**LGC14.2 1vs4**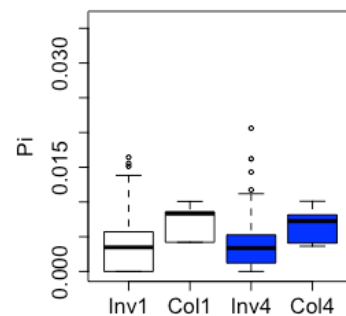**LGC14.2 2vs4**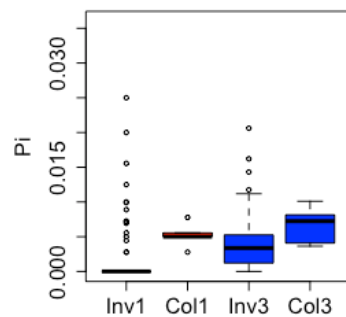**LGC14.3**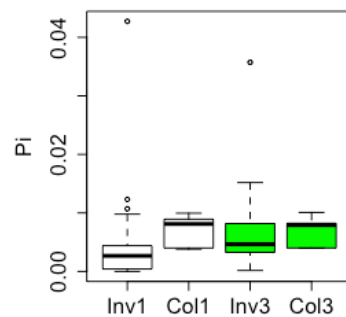

Supplement: Supplementary file 5 [file MEC-28-1375-s005.pdf]

**LGC1.1**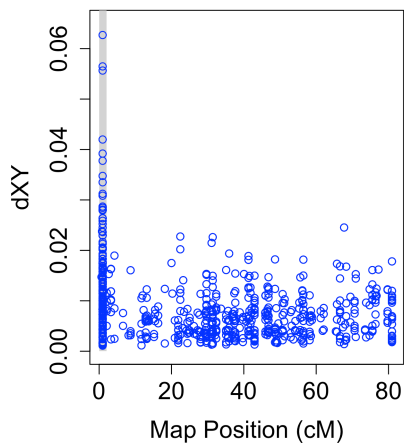**LGC1.2**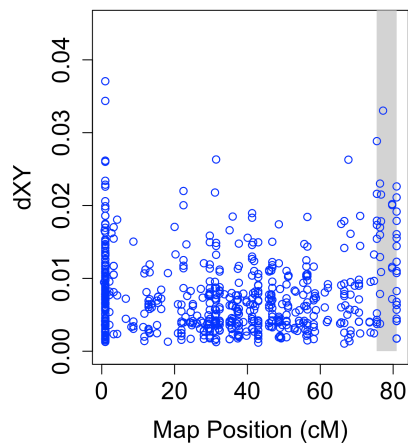**LGC2.1**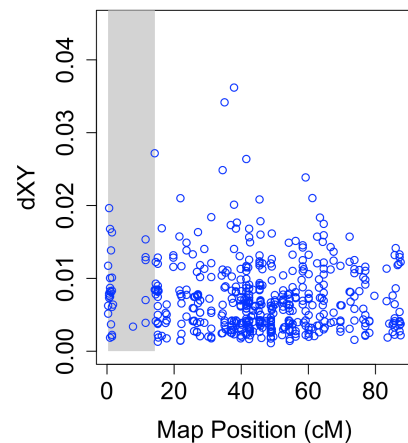**LGC4.1**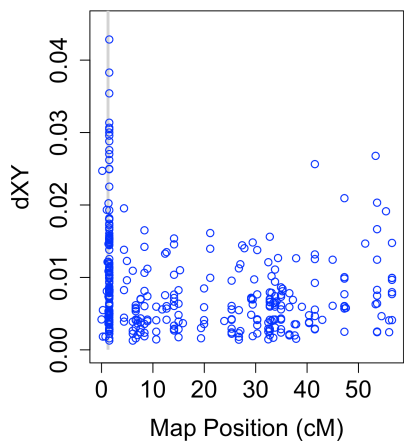**LGC6.1**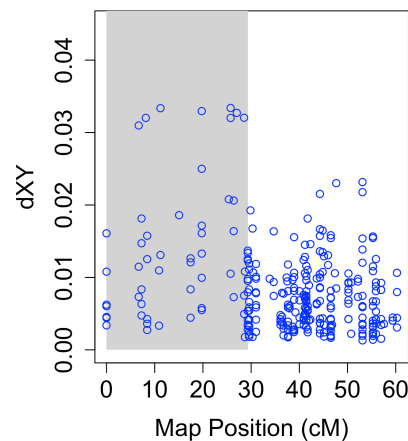**LGC6.2 6vs4**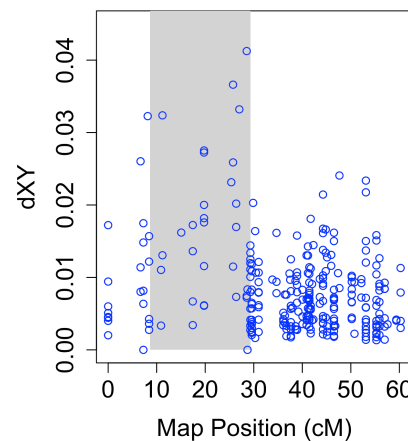**LGC6.2 6vs2**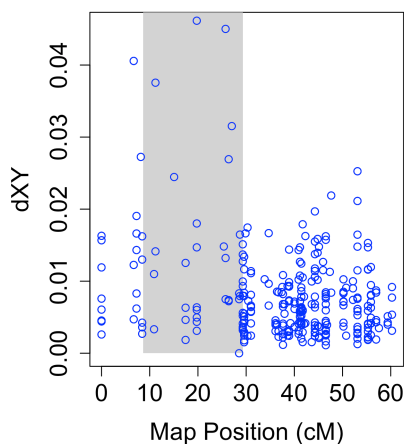**LGC6.2 4vs2**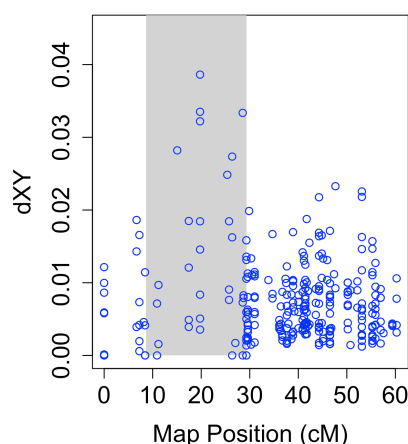**LGC7.1**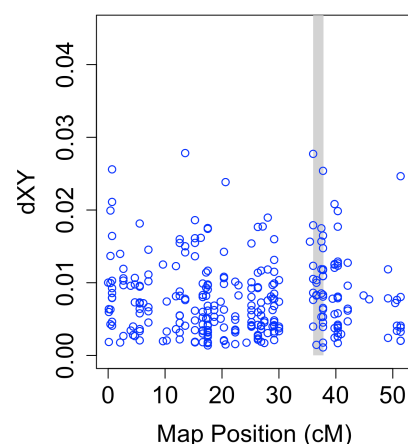**LGC7.2**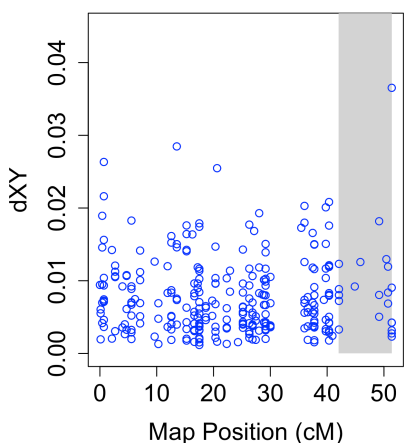**LGC9.1**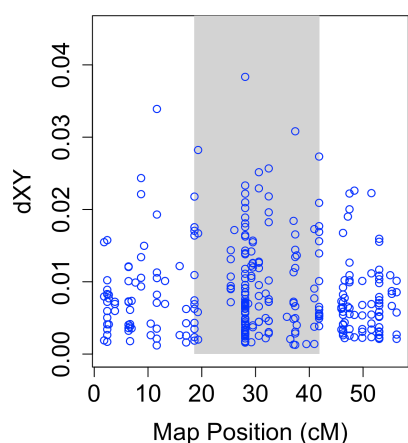**LGC10.1**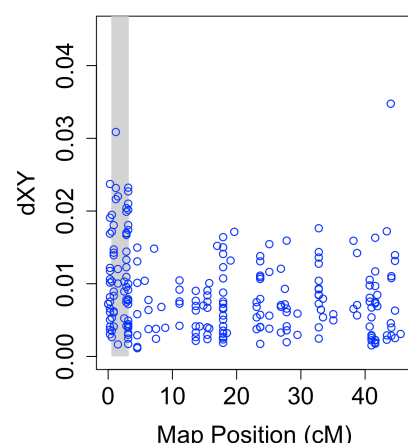

**LGC11.1**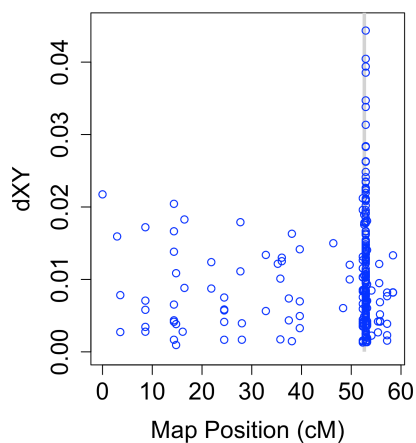**LGC12.1**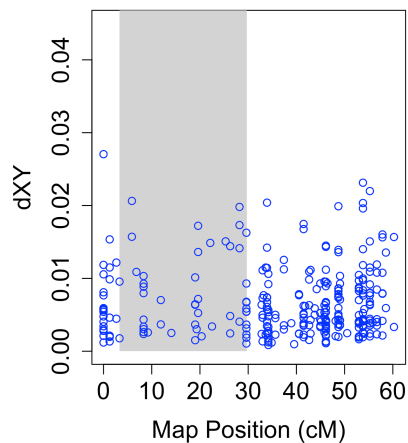**LGC12.2**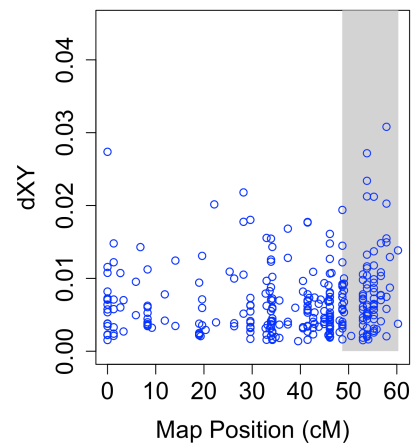**LGC14.1**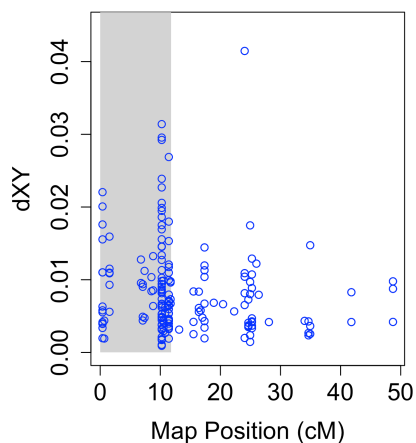**LGC14.2 2vs1**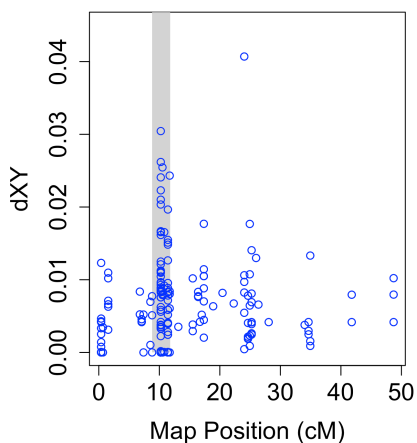**LGC14.2 4vs1**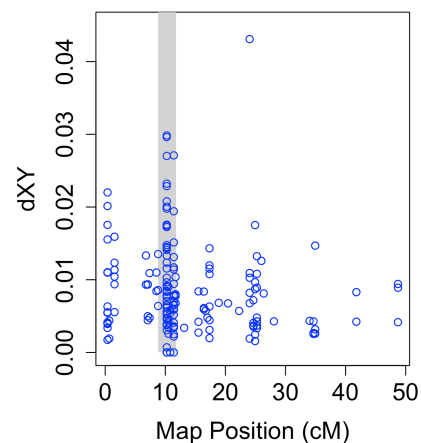**LGC14.2 4vs2**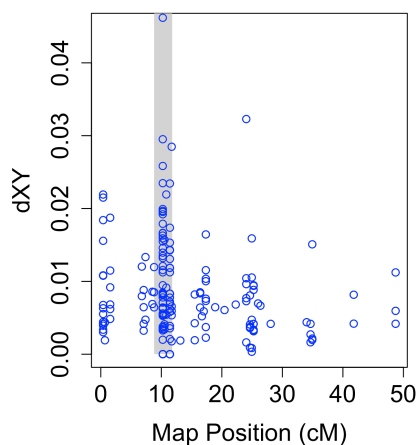**LGC14.3**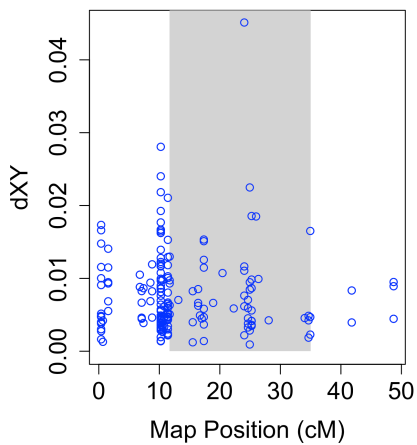

Supplement: Supplementary file 6 [file MEC-28-1375-s006.pdf]

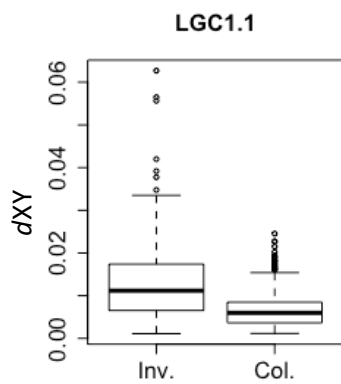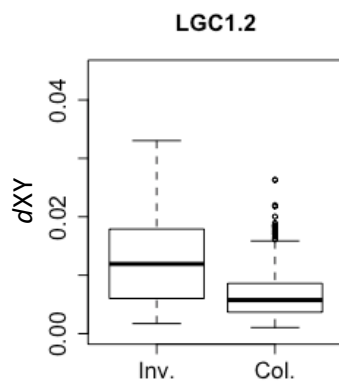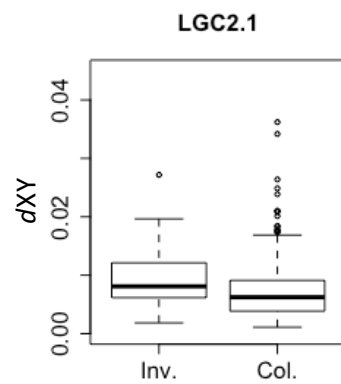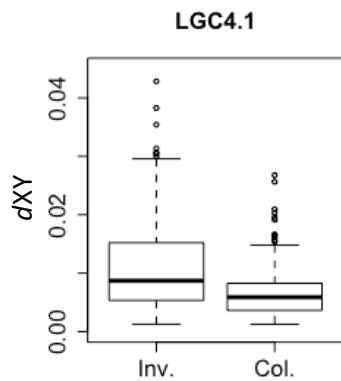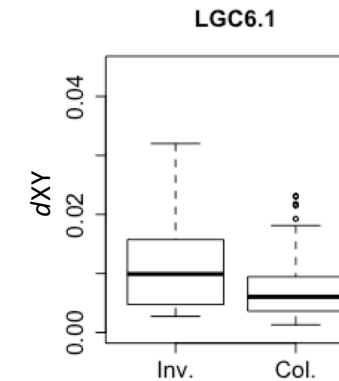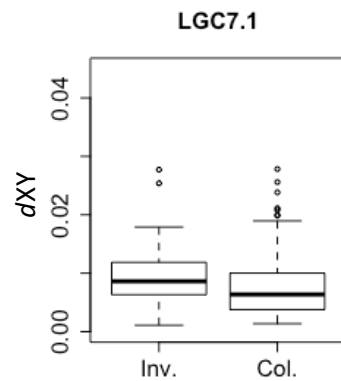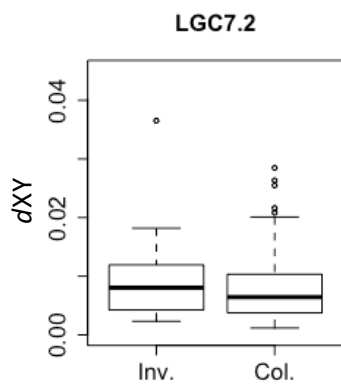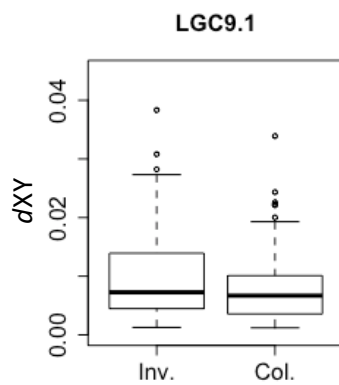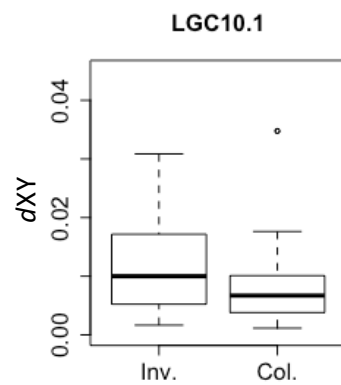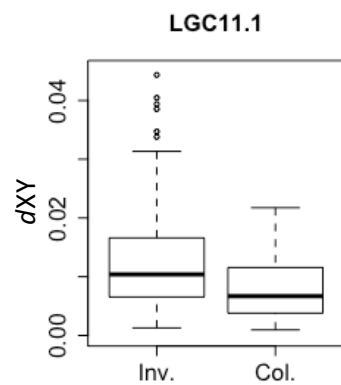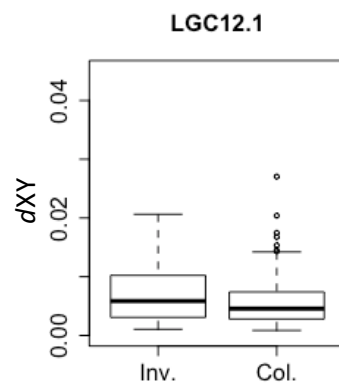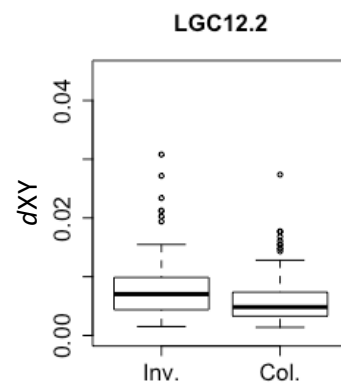

**LGC14.1**

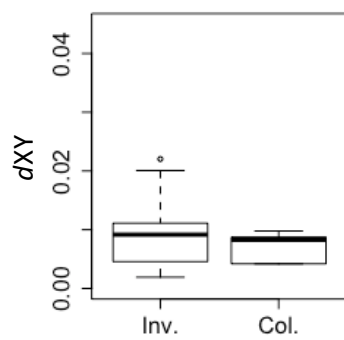

**LGC14.2 4vs2**

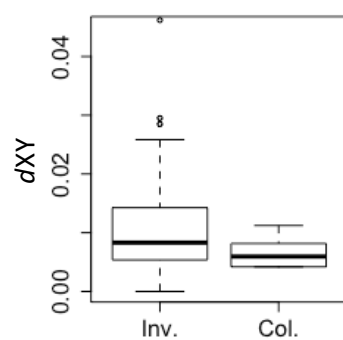

**LGC14.2 2vs1**

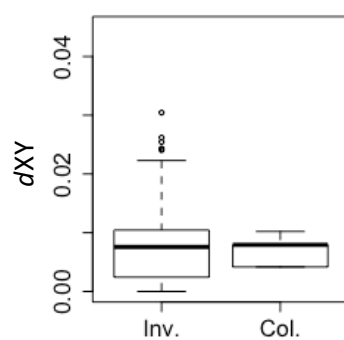

**LGC14.2 4vs1**

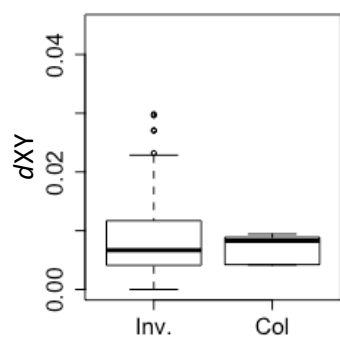

**LGC14.3**

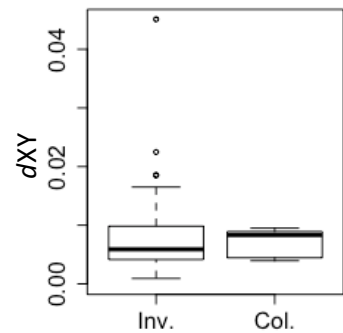

Supplement: Supplementary file 7 [file MEC-28-1375-s007.pdf]
