# Supplementary material for: Multiple chromosomal rearrangements in a hybrid zone between Littorina saxatilis ecotypes
Source: Mol Ecol. 2019 Feb 25;28(6):1375–93. doi: 10.1111/mec.14972 (PMC6518922; doi:10.1111/mec.14972)
Supplement: Supplementary file 2 [file MEC-28-1375-s002.pdf]

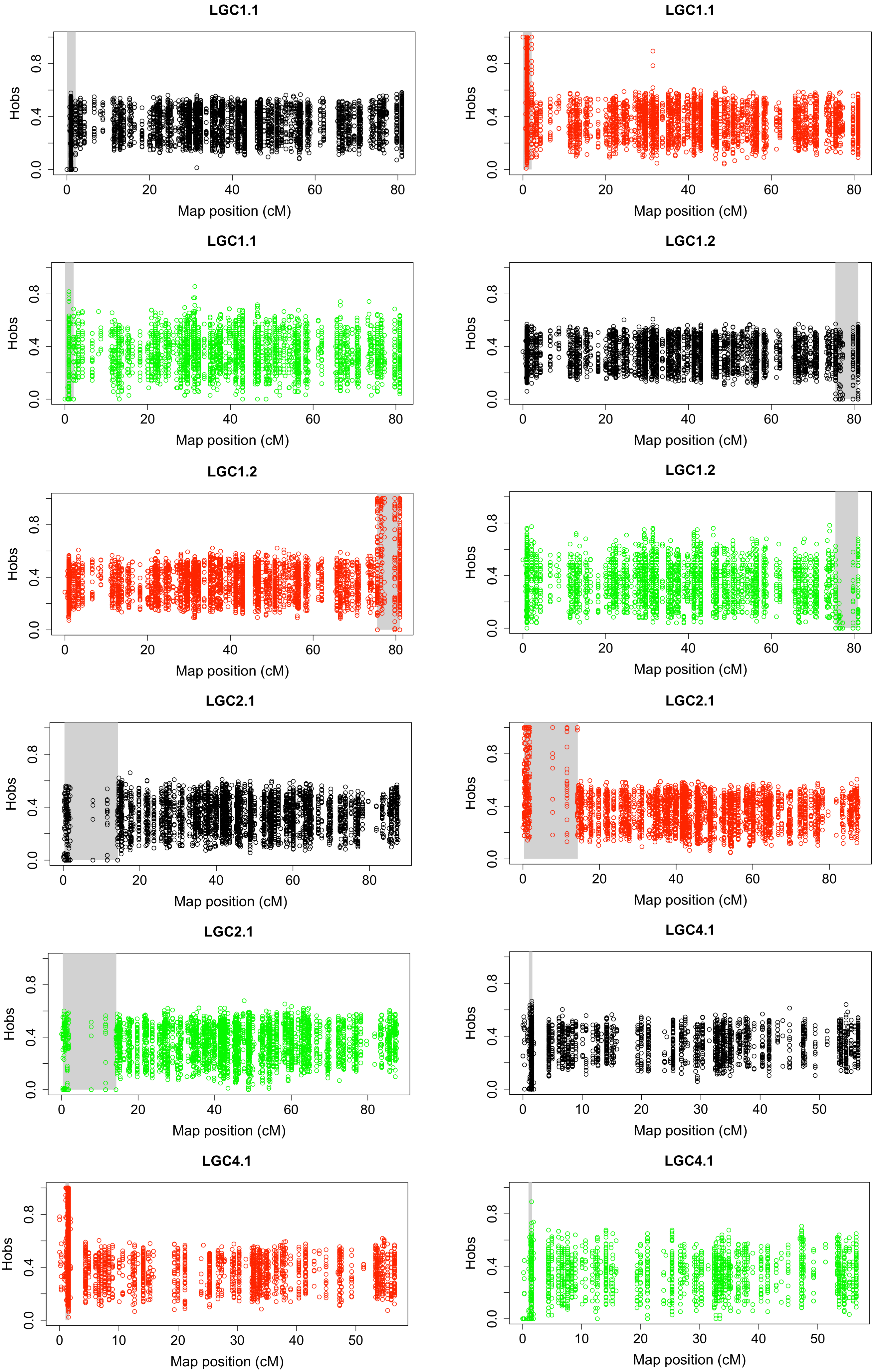
LGC1.1
LGC1.1
LGC1.1
LGC1.2
LGC1.2
LGC1.2
LGC2.1
LGC2.1
LGC2.1
LGC4.1
LGC4.1
LGC4.1
Hobs
0.0
0.4
0.8
Map position (cM)
0
10
20
30
40
50
60
80

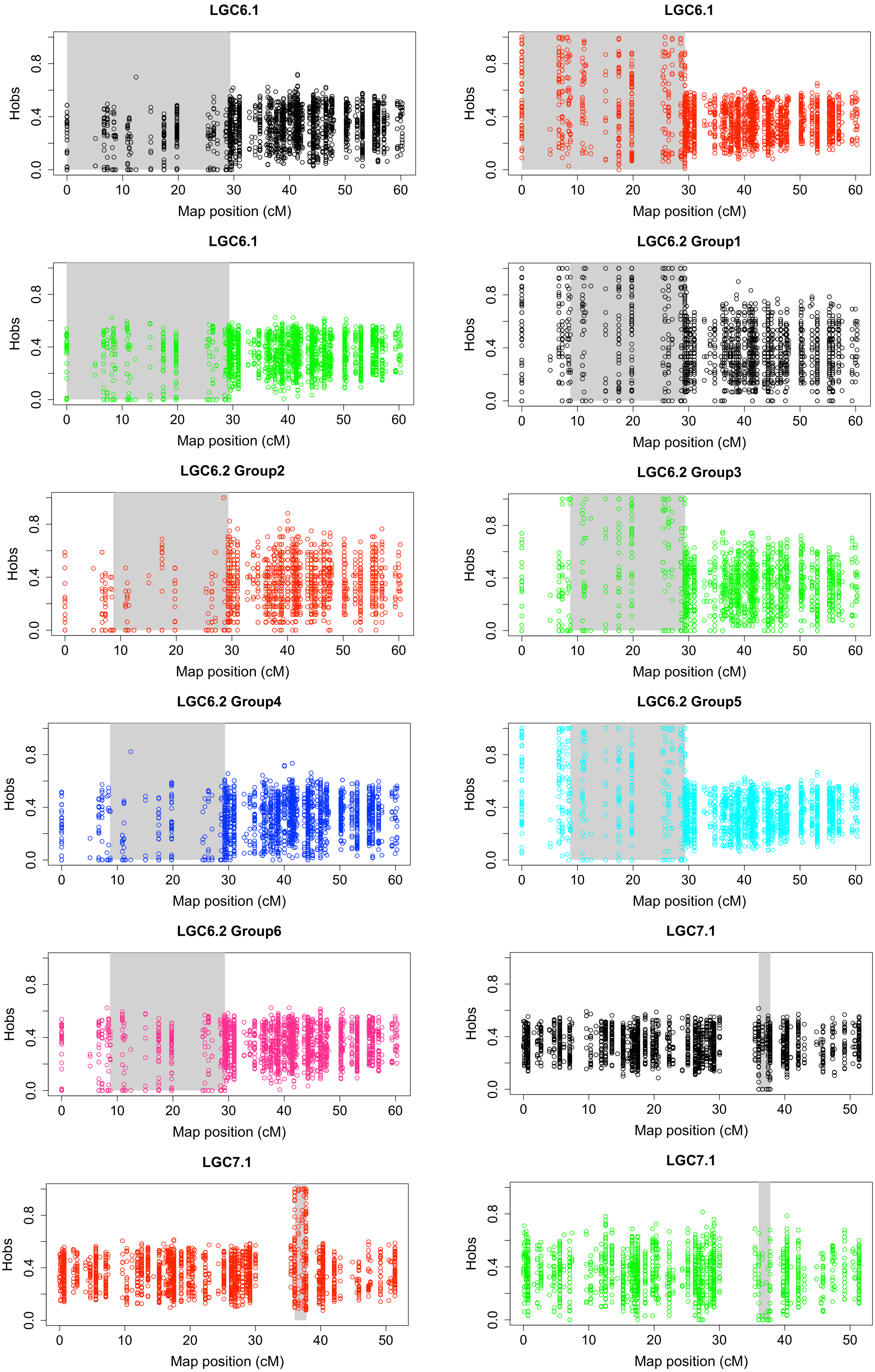
LGC6.1
Hobs
Map position (cM)
LGC6.1
Hobs
Map position (cM)
LGC6.1
Hobs
Map position (cM)
LGC6.2 Group1
Hobs
Map position (cM)
LGC6.2 Group2
Hobs
Map position (cM)
LGC6.2 Group3
Hobs
Map position (cM)
LGC6.2 Group4
Hobs
Map position (cM)
LGC6.2 Group5
Hobs
Map position (cM)
LGC6.2 Group6
Hobs
Map position (cM)
LGC7.1
Hobs
Map position (cM)
LGC7.1
Hobs
Map position (cM)
LGC7.1
Hobs
Map position (cM)

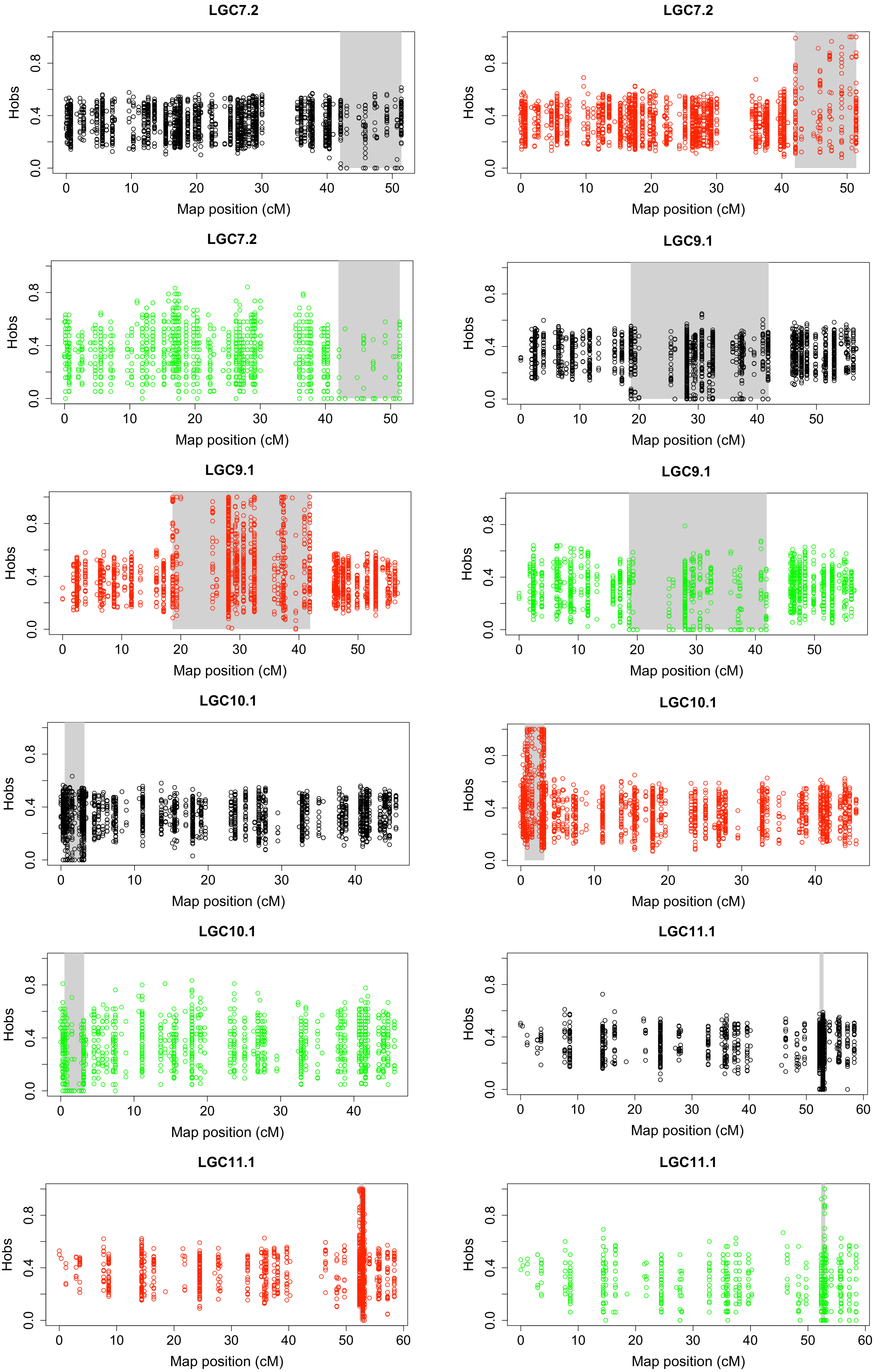
LGC7.2
Hobs
Map position (cM)
LGC7.2
Hobs
Map position (cM)
LGC7.2
Hobs
Map position (cM)
LGC9.1
Hobs
Map position (cM)
LGC9.1
Hobs
Map position (cM)
LGC9.1
Hobs
Map position (cM)
LGC10.1
Hobs
Map position (cM)
LGC10.1
Hobs
Map position (cM)
LGC10.1
Hobs
Map position (cM)
LGC11.1
Hobs
Map position (cM)
LGC11.1
Hobs
Map position (cM)
LGC11.1
Hobs
Map position (cM)

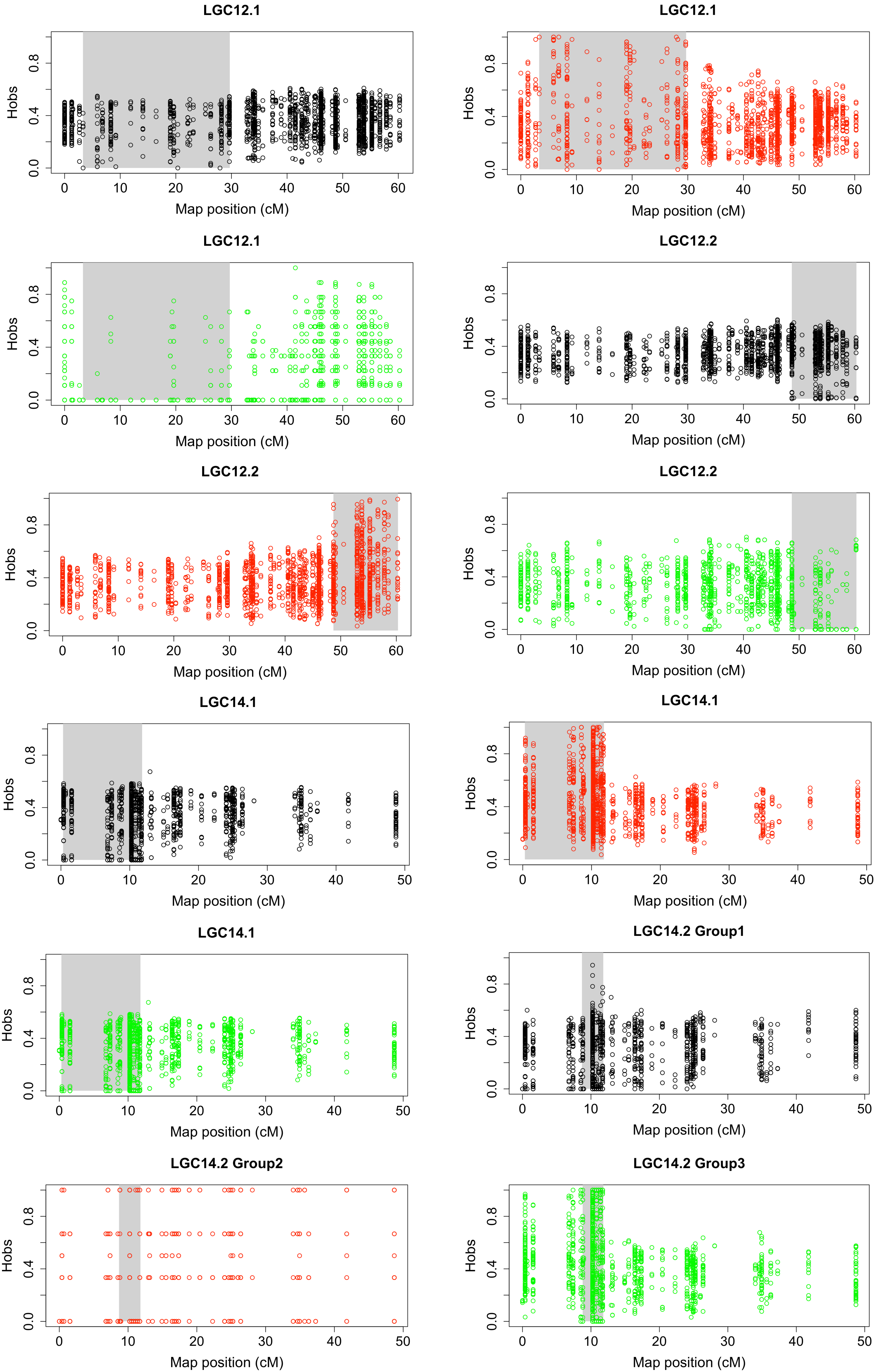

LGC12.1
LGC12.1
LGC12.1
LGC12.2
LGC12.2
LGC12.2
LGC14.1
LGC14.1
LGC14.1
LGC14.2 Group1
LGC14.2 Group2
LGC14.2 Group3
Hobs
0.0
0.4
0.8
Map position (cM)
0
10
20
30
40
50
60

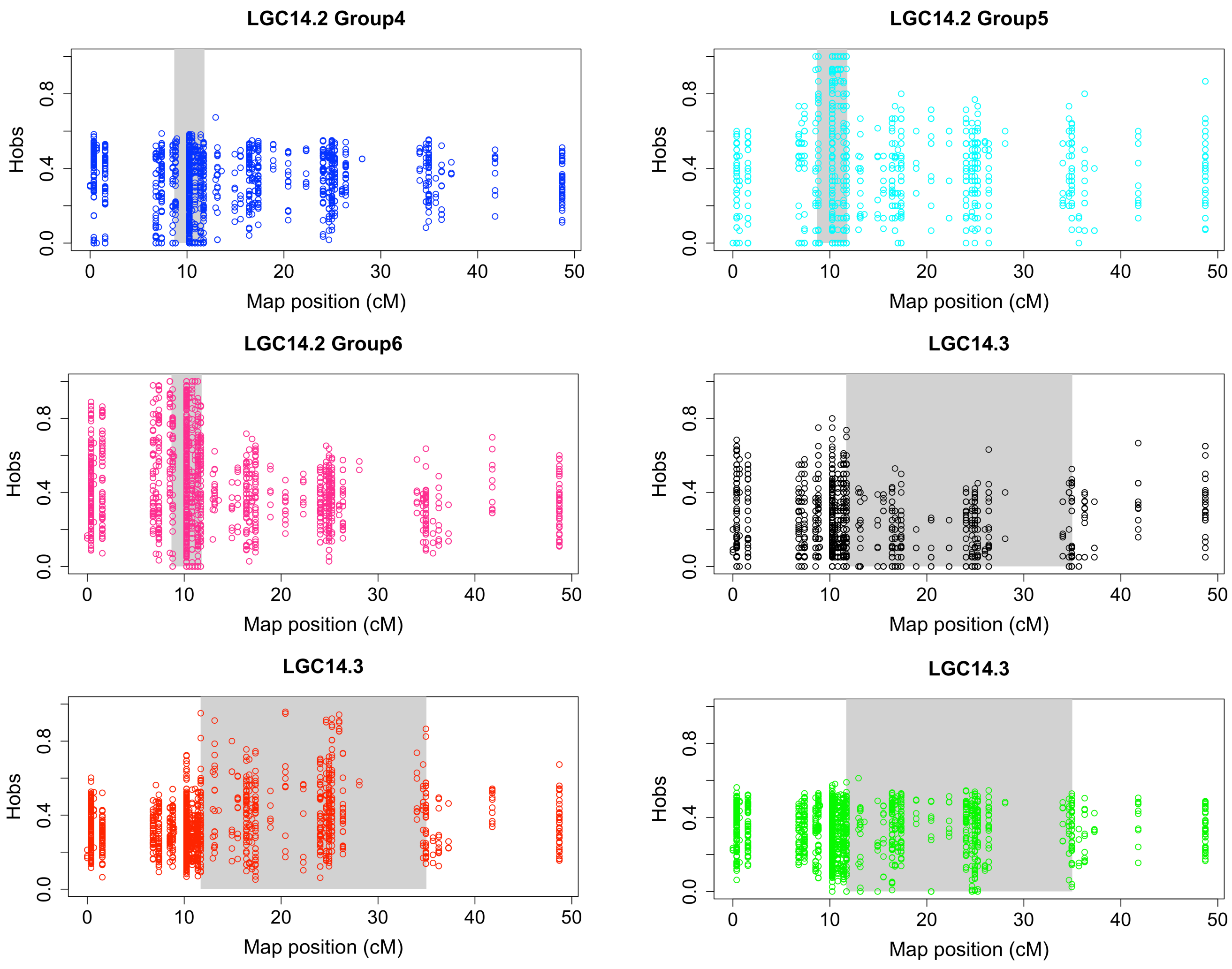
LGC14.2 Group4
LGC14.2 Group5
LGC14.2 Group6
LGC14.3
LGC14.3
LGC14.3
Hobs
0.0
0.4
0.8
Map position (cM)
0
10
20
30
40
50
