# Supplementary material for: Multiple chromosomal rearrangements in a hybrid zone between Littorina saxatilis ecotypes
Source: Mol Ecol. 2019 Feb 25;28(6):1375–93. doi: 10.1111/mec.14972 (PMC6518922; doi:10.1111/mec.14972)
Supplement: Supplementary file 4 [file MEC-28-1375-s004.pdf]

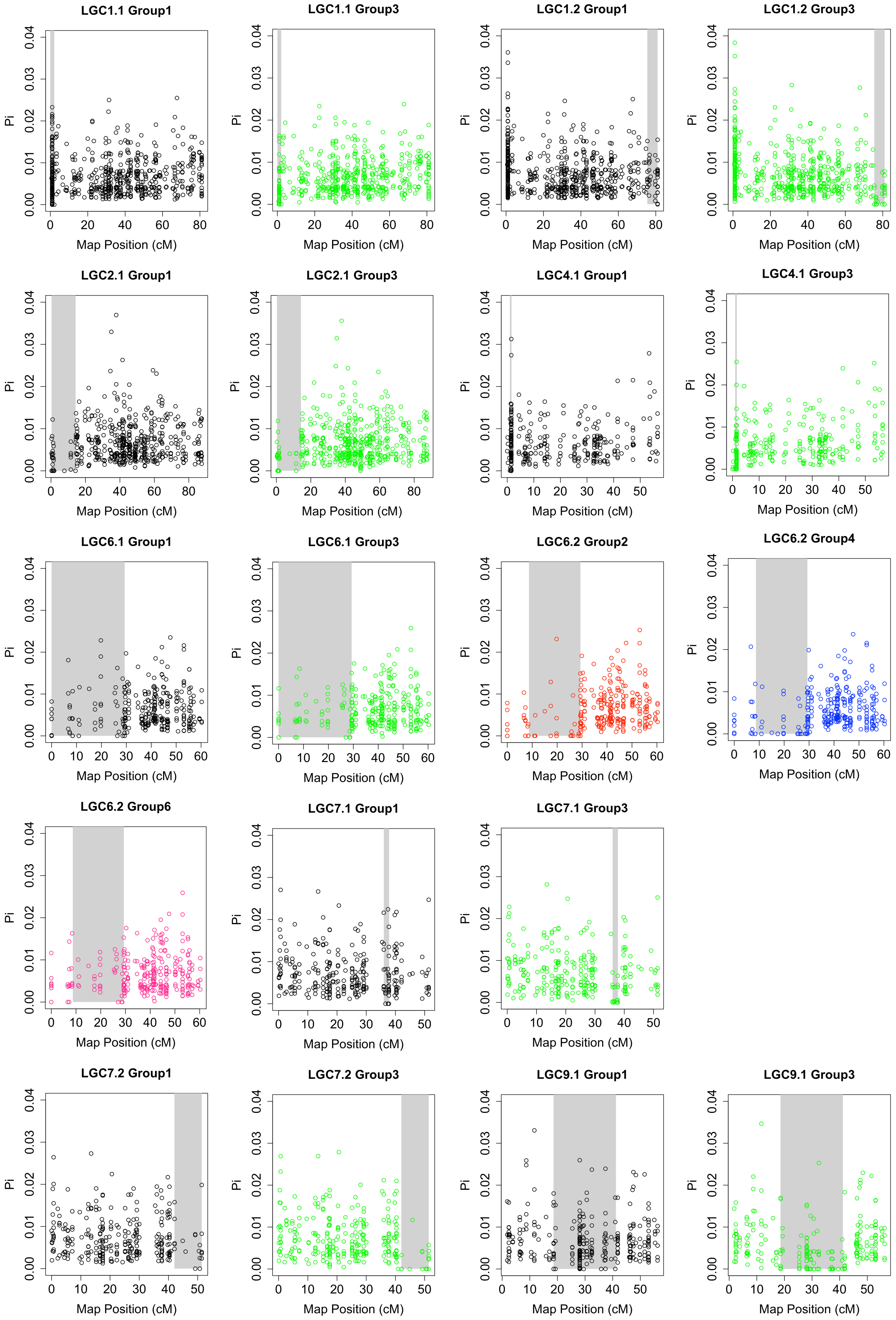
LGC1.1 Group1
LGC1.1 Group3
LGC1.2 Group1
LGC1.2 Group3
LGC2.1 Group1
LGC2.1 Group3
LGC4.1 Group1
LGC4.1 Group3
LGC6.1 Group1
LGC6.1 Group3
LGC6.2 Group2
LGC6.2 Group4
LGC6.2 Group6
LGC7.1 Group1
LGC7.1 Group3
LGC7.2 Group1
LGC7.2 Group3
LGC9.1 Group1
LGC9.1 Group3
Pi
Map Position (cM)

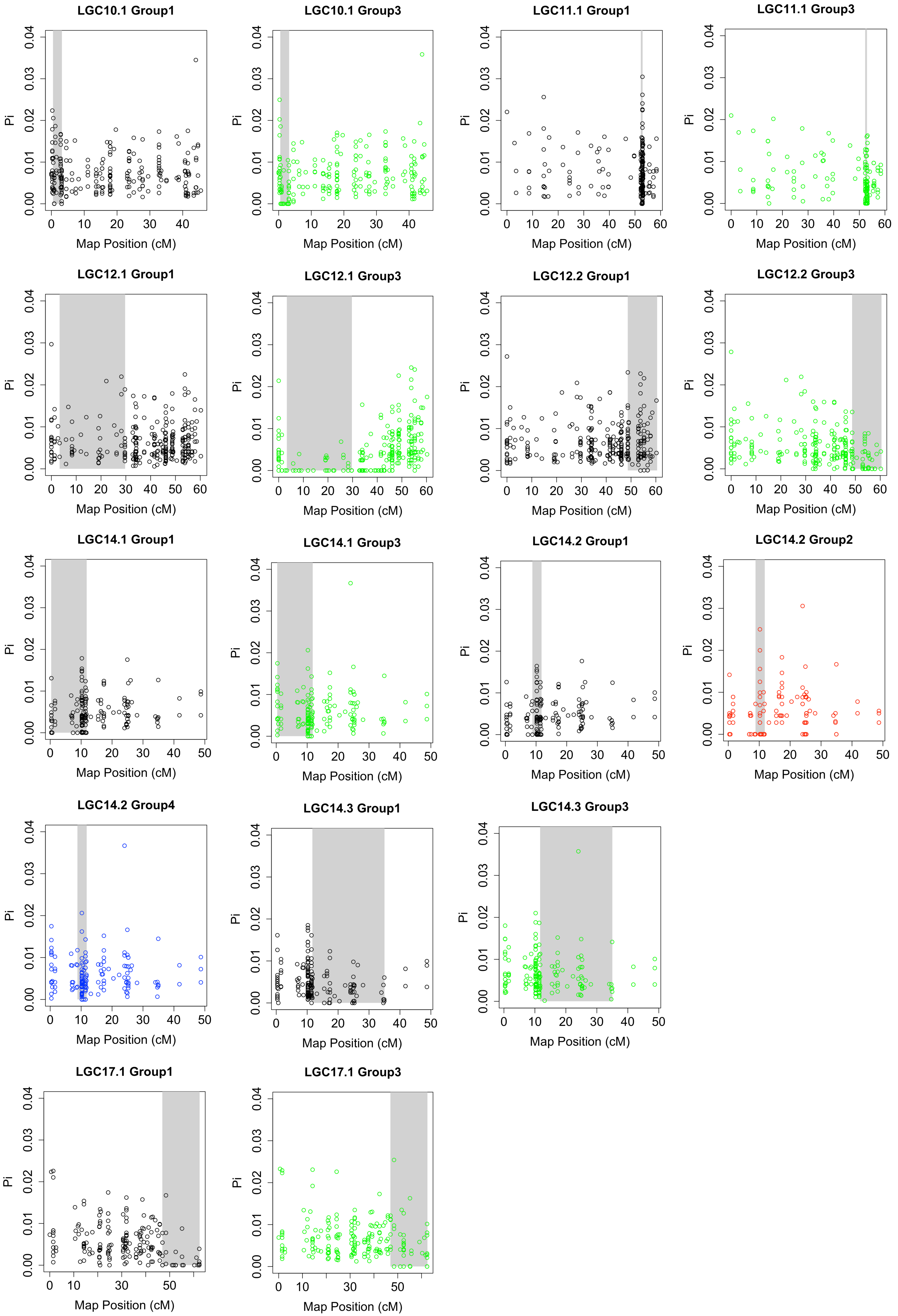
LGC10.1 Group1
LGC10.1 Group3
LGC11.1 Group1
LGC11.1 Group3
LGC12.1 Group1
LGC12.1 Group3
LGC12.2 Group1
LGC12.2 Group3
LGC14.1 Group1
LGC14.1 Group3
LGC14.2 Group1
LGC14.2 Group2
LGC14.2 Group4
LGC14.3 Group1
LGC14.3 Group3
LGC17.1 Group1
LGC17.1 Group3
Pi
Map Position (cM)
